# Supplementary material for: Whole-Genome and RNA Sequencing Reveal Variation and Transcriptomic Coordination in the Developing Human Prefrontal Cortex
Source: Cell Rep. Author manuscript; Available in PMC 2020 Jun 15. (PMC7295160; doi:10.1016/j.celrep.2020.03.053)
Supplement: 1 [file NIHMS1582939-supplement-1.pdf]

Cell Reports, Volume *31*

Supplemental Information

Whole-Genome and RNA Sequencing Reveal Variation and Transcriptomic Coordination in the Developing Human Prefrontal Cortex

Donna M. Werling, Sirisha Pochareddy, Jinmyung Choi, Joon-Yong An, Brooke Sheppard, Minshi Peng, Zhen Li, Claudia Dastmalchi, Gabriel Santpere, André M.M. Sousa, Andrew T.N. Tebbenkamp, Navjot Kaur, Forrest O. Gulden, Michael S. Breen, Lindsay Liang, Michael C. Gilson, Xuefang Zhao, Shan Dong, Lambertus Klei, A. Ercument Cicek, Joseph D. Buxbaum, Homa Adle-Biassette, Jean-Leon Thomas, Kimberly A. Aldinger, Diana R. O'Day, Ian A. Glass, Noah A. Zaitlen, Michael E. Talkowski, Kathryn Roeder, Matthew W. State, Bernie Devlin, Stephan J. Sanders, and Nenad Sestan

Supplemental Information

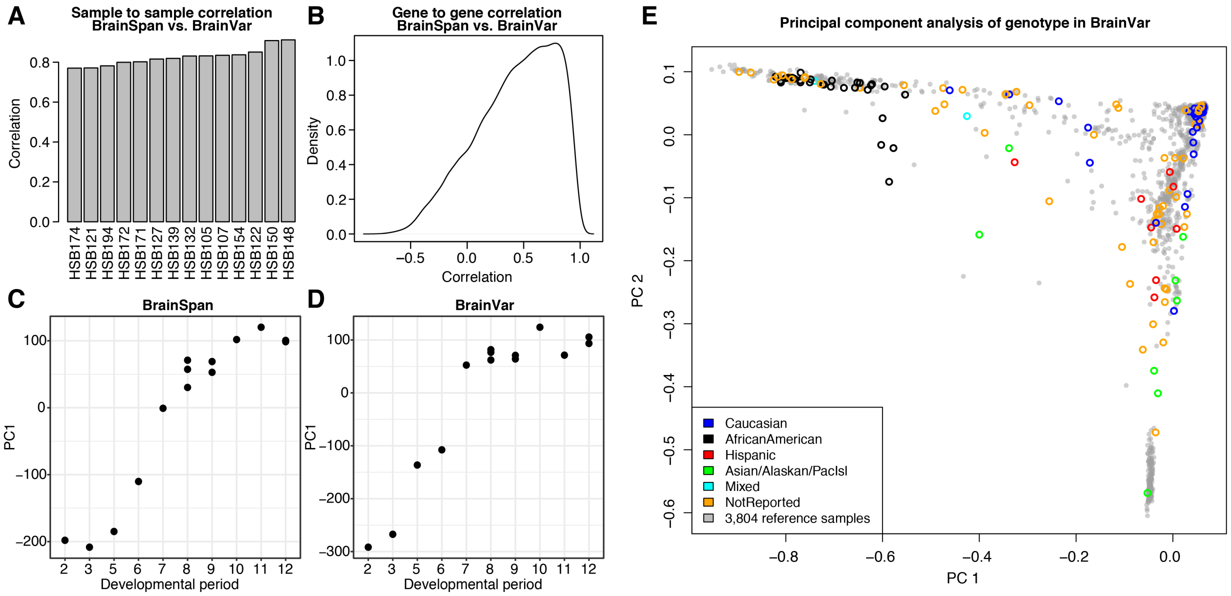

Figure S1. Comparison of RNA-seq data between BrainSpan and BrainVar and WGS ancestry prediction, related to Figures 1, 2. A) Pearson correlation coefficients of gene expression for 14 overlapping samples with RNA-seq data generated from the dorsolateral frontal cortex in BrainSpan (Li et al., 2018) and BrainVar. **B)** Distribution of Pearson correlation coefficients between 23,782 cortically expressed genes for RNA-seq data for 14 overlapping samples in BrainSpan (Li et al., 2018) and BrainVar. **C)** First principal component of gene expression by developmental period for all samples in the BrainSpan dataset (Li et al., 2018). **D)** First principal component of gene expression by developmental period for all samples in BrainVar. **E)** Principal component analysis using common variation called from WGS data for all 176 samples in BrainVar against a reference of 3,804 independent parents with WGS data from the Simons Simplex Collection (An et al., 2018). Self-reported ancestry is indicated by color for the BrainVar samples.

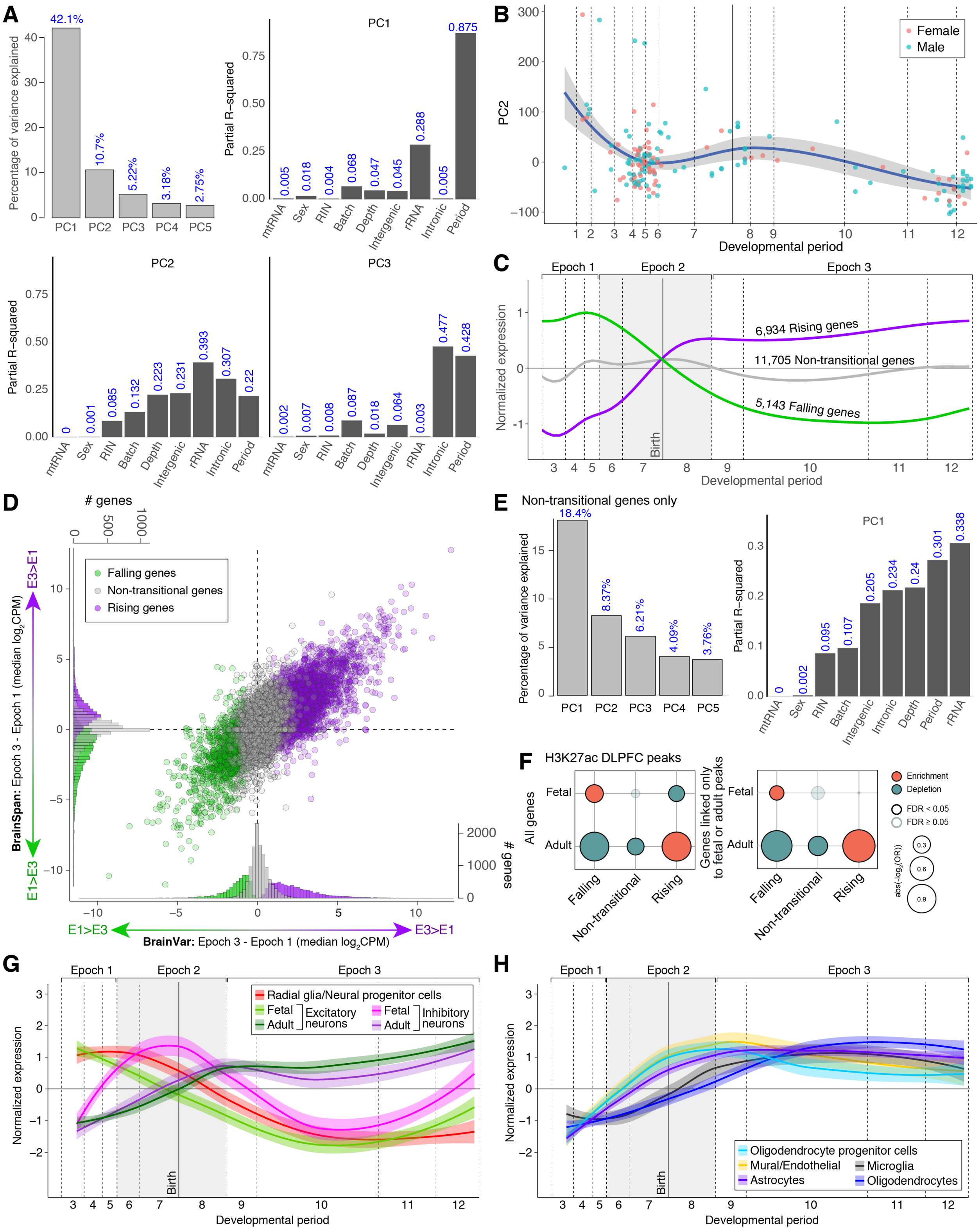

Figure S2. Principal component analysis of gene expression, related to Figure 2. A) A scree plot showing the variance in gene expression explained by the first five principal components (PC1 to PC5) across all samples and 23,782 cortically-expressed genes in the BrainVar dataset. **B)** The relationship between PC2 (y-axis) and

developmental period (x-axis); the equivalent plot for PC1 is shown in Figure 2A of the main manuscript. **C)** Relationship between PC1, PC2, and PC3 for each sample (points) with developmental period indicated by color and genotypic sex by symbol. **D)** Correlation between PC1, PC2, and PC3, known variables, and residuals. **E)** Trajectory analysis identified 12,077 genes involved in the late-fetal transition and 11,705 Non-transitional genes (Figure 2B). To assess the extent to which the late-fetal transition explains the temporal variance captured by PC1 in the initial analysis, we repeated the principal component analysis for the 11,705 Non-transitional genes. PC1 of this secondary analysis explains on 18.4% of the variance in gene expression. **F)** The correlation between PC1 and known variables and residuals for the secondary analysis based on Non-transitional genes only. **G)** The enrichment for H3K27ac peaks, detected in the fetal and adult human dorsolateral frontal cortex in BrainSpan (Li et al., 2018), with Falling, Non-transitional and Rising genes. The analysis is shown for all 23,782 cortically-expressed genes (top) and limited to genes with an associated H3K27ac peak during at least one developmental stage.

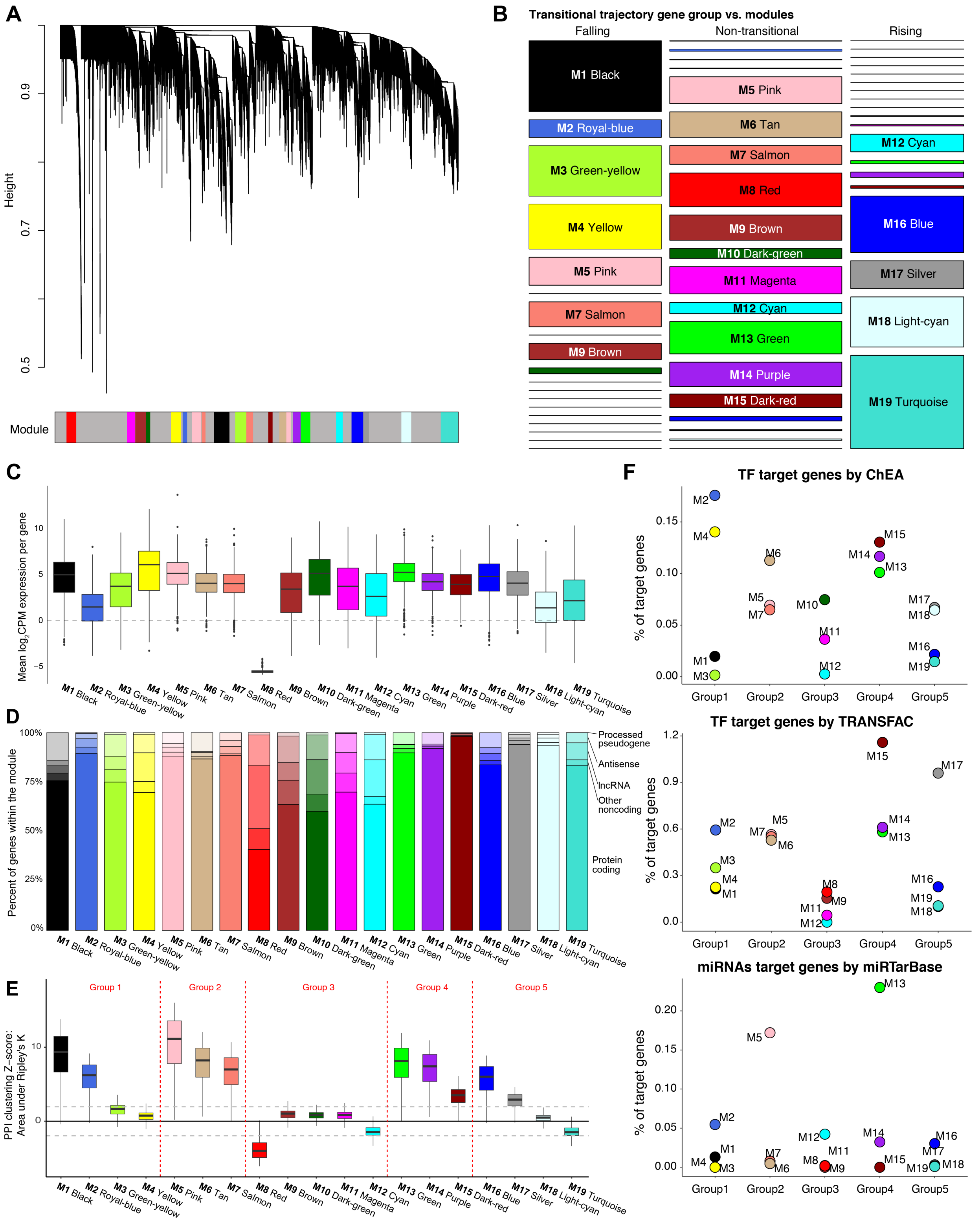

Figure S3. WGCNA module relationship and characteristics, related to Figure 3. A) Dendrogram of 10,459 cortically-expressed genes assigned to 19 WGCNA modules; 13,323 genes were not assigned to any module (grey). **B)** A mosaic plot showing the overlap of genes between modules (y-axis and color) and the three transitional

trajectory gene sets (x-axis), with the area of each rectangle representing the number of overlapping genes. **C)** Median log_2CPM expression of genes within each module (x-axis and color) across all 176 samples in the cohort. **D)** The percentage of protein-coding and noncoding genes (lncRNA, Antisense, Processed pseudogenes, and other noncoding shown by opacity) within each module. **E)** The connectivity within BioGRID protein-protein interaction (PPI) networks of the genes within each module is shown as a Z-score distribution (Cornish and Markowetz, 2014) by permutation against all 23,782 cortically-expressed genes. **F)** The percentage of genes in each module targeted by transcription factors (TFs) predicted by ChIP Enrichment Analysis (ChEA, (Lachmann et al., 2010), top) and TRANSFAC (http://genexplain.com/transfac/, middle) and the percentage of genes targeted by miRNA prediction from the mirTarbase database ((Chou et al., 2018), bottom).

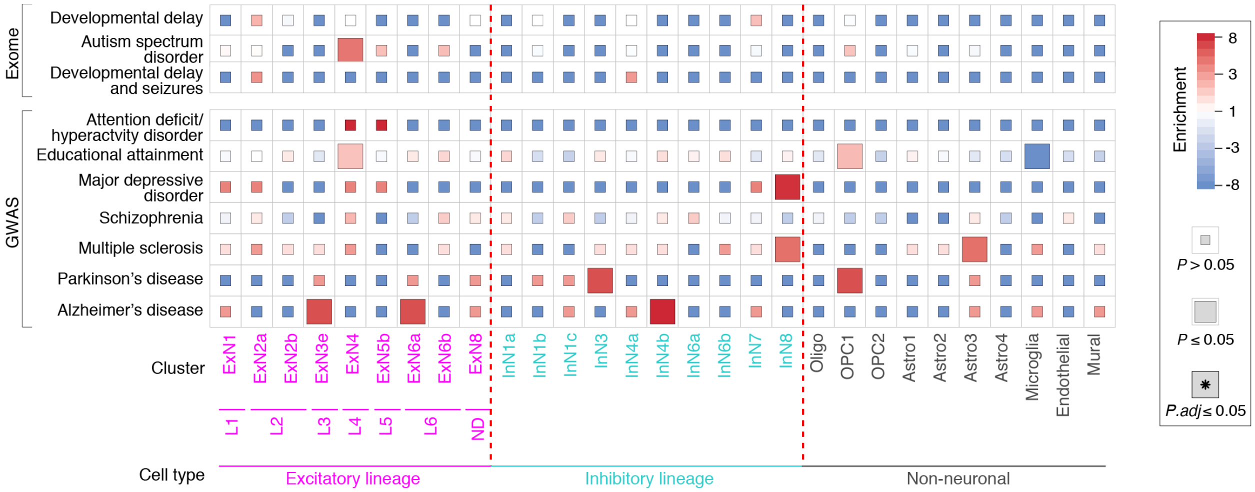

Figure S4. Expression of genes associated with CNS traits and disorders, related to Figure 4. The enrichment of genes associated with ten CNS traits and disorders (Figure 4) is shown in cell type clusters from single-cell sequencing of three adult human brain samples (Li et al., 2018). Abbreviations: C: cluster of single nuclei; L: cortical layer; ND: layer not defined; RG: radial glia; IPC: intermediate progenitor cell; NN: newborn neuron; ExN: excitatory neuron; InN: inhibitory neuron; CGE: caudal ganglionic eminence; MGE: medial ganglionic eminence; OPC: oligodendrocyte progenitor cell. Statistical analysis: FET, corrected for 290 comparisons.

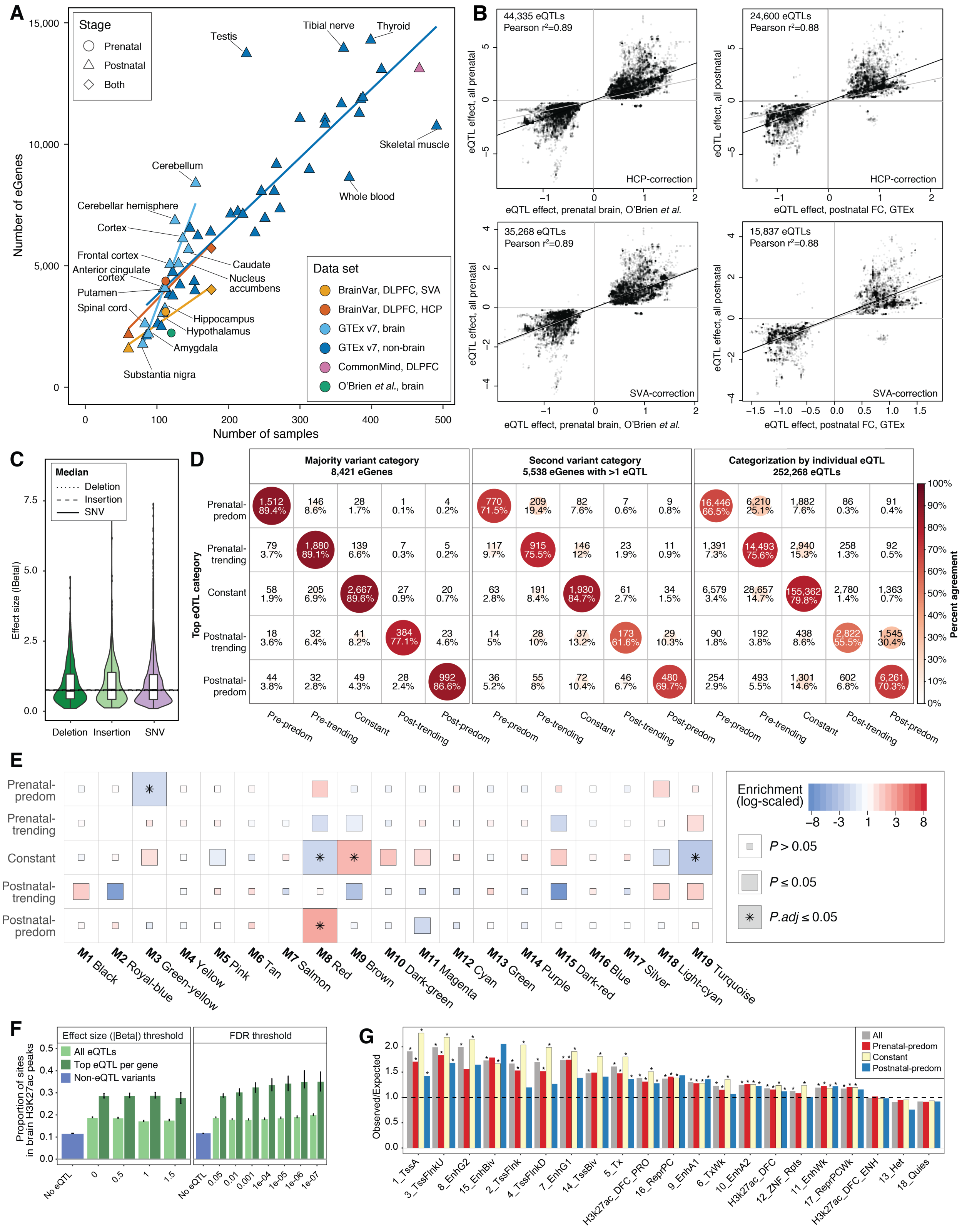

Figure S5. eGene concordance and characteristics, related to Figure 5. A) Scatterplot and best fit lines for the number of eGenes (y-axis) against sample size (x-axis) reported in BrainVar and several published data sets (Fromer

et al., 2016; O'Brien et al., 2018; The GTEx Consortium et al., 2017). **B)** Scatterplot of the eQTL direction and magnitude of effect for variant-gene pairs reaching FDR-significance in BrainVar and published datasets (O'Brien et al., 2018; The GTEx Consortium et al., 2017). The plots on the left compare BrainVar to prenatal whole brain (O'Brien et al., 2018), while those on the right compare BrainVar to adult DLPFC (The GTEx Consortium et al., 2017). The plots at the top are for eQTLs discovered in the BrainVar prenatal samples, while those at the bottom are eQTLs discovered in BrainVar postnatal samples. Best fit line is plotted in black, slope of 1 is plotted in gray. **C)** Distribution of the absolute value of eQTL effect size (regression beta) for all eQTLs binned by deletions, insertions, and single nucleotide variants (SNV). **D)** The temporal predominance of an eGene was defined by the eQTL with the lowest p-value (top eQTL). To assess the consistency of this approach, we assessed the number of eGenes where the temporal predominance of the top eQTL matched the majority of eQTLs for the eGene (left) or the eQTL with the second lower p-value (right). **E)** Enrichment of 19 WGCNA modules with eGenes, divided into five categories based on temporal specificity. **F)** Proportion of eQTLs that overlap an H3K27ac peak detected in human DLPFC in BrainSpan (Li et al., 2018). Results are binned by eQTL effect size (left) and FDR threshold (right) showing all eQTLs and the top eQTL per gene (shown by shade). **G)** Barplot of observed versus expected overlap using GREGOR analysis between Temporal-predominant and Constant eQTLs with functional loci from 18 chromatin states defined by the Roadmap Epigenome Project (https://egg2.wustl.edu/roadmap/web_portal/chr_state_learning.html#exp_18state).

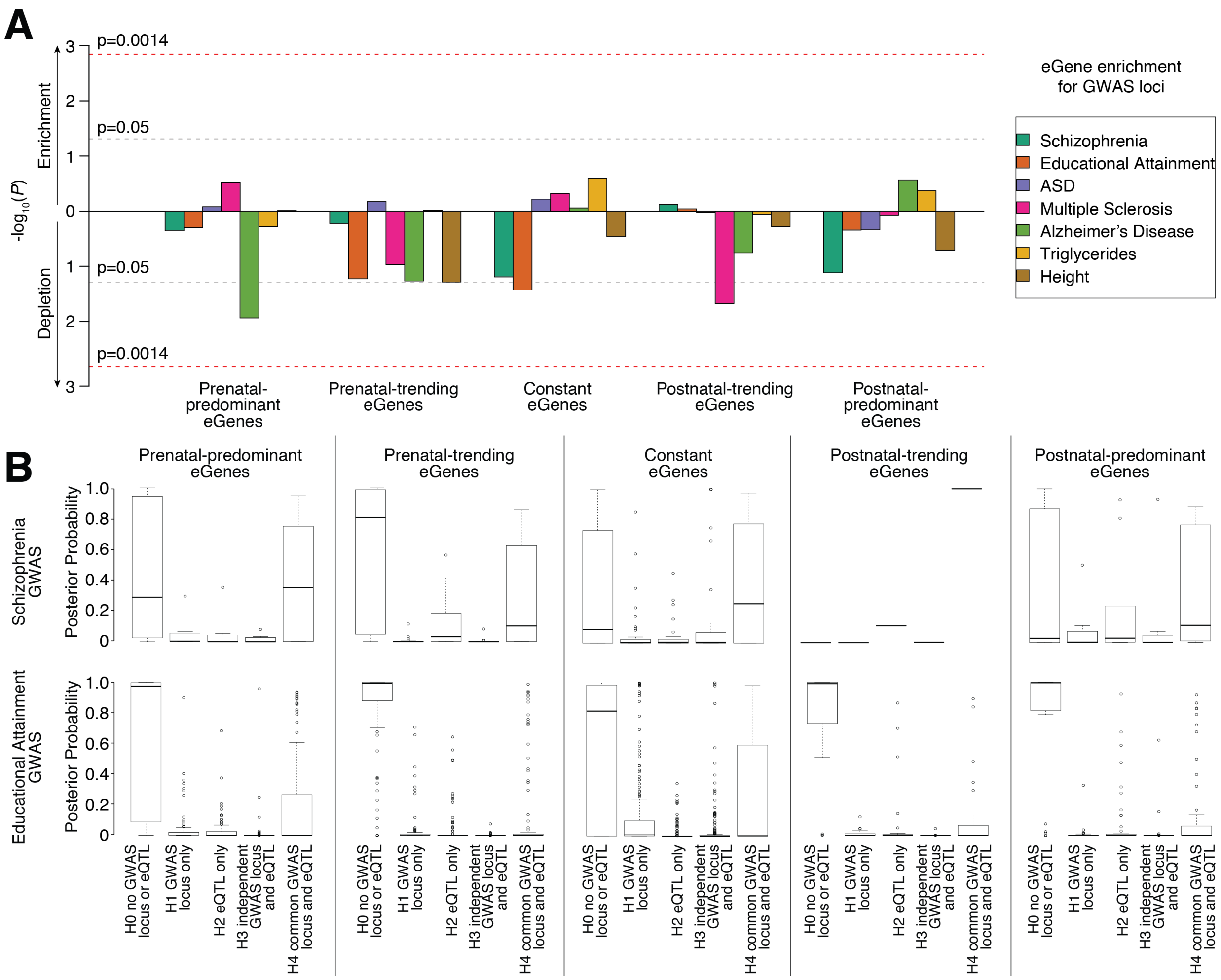

Figure S6. eQTL enrichments and colocalization, related to Figure 6. A) Barplot of enrichment p-values for GWAS signal in temporal categories of eGenes using MAGMA analysis. Red dashed line indicates significance threshold after Bonferroni correction for tests of 7 GWAS phenotypes and 5 eGene categories. **B)** Boxplots of posterior probabilities (PPs) for tests of colocalization between eQTL and GWAS loci. For each tested region, the PP is shown for the 5 hypotheses tested using Coloc analysis software, grouped by temporal category of the eGenes associated with the eQTL loci.
