# Supplementary material for: Whole-Genome and RNA Sequencing Reveal Variation and Transcriptomic Coordination in the Developing Human Prefrontal Cortex
Source: Cell Rep. Author manuscript; Available in PMC 2020 Jun 15. (PMC7295160; doi:10.1016/j.celrep.2020.03.053)
Supplement: 8 [file NIHMS1582939-supplement-8.pdf]

Resource

Cell Reports

Whole-Genome and RNA Sequencing Reveal Variation and Transcriptomic Coordination in the Developing Human Prefrontal Cortex

Graphical Abstract

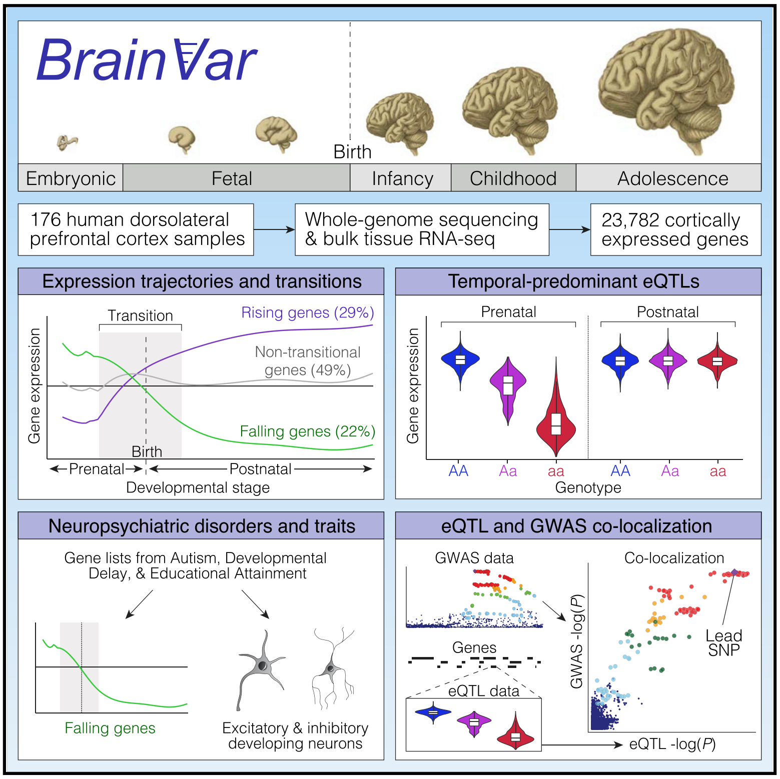

Highlights

- Whole-genome and RNA sequencing across human prefrontal cortex development
- Gene-specific developmental trajectories characterize the late-fetal transition
- Identification of constant, prenatal-predominant, and postnatal-predominant eQTLs
- Integrated analysis implicates genes in loci associated with educational attainment

Authors

Donna M. Werling, Sirisha Pochareddy, Jinmyung Choi, ..., Bernie Devlin, Stephan J. Sanders, Nenad Sestan

Correspondence

stephan.sanders@ucsf.edu (S.J.S.),
nenad.sestan@yale.edu (N.S.)

In Brief

Werling et al. analyze gene expression across the span of human cerebral cortical development and profile the trajectories of individual genes, coordinated groups of genes, and their relationships to disorders. Integration of genetic variation identifies quantitative trait loci that implicate specific genes in loci associated with neuropsychiatric traits and disorders.

Werling et al., 2020, Cell Reports *31*, 107489
April 7, 2020 © 2020 The Author(s).
https://doi.org/10.1016/j.celrep.2020.03.053

CellPress

Cell Reports
Resource

OPEN ACCESS
CellPress

Whole-Genome and RNA Sequencing Reveal Variation and Transcriptomic Coordination in the Developing Human Prefrontal Cortex

Donna M. Werling,[1,2,32] Sirisha Pochareddy,[3,32] Jinmyung Choi,[3,32] Joon-Yong An,[1,4,5,32] Brooke Sheppard,[1] Minshi Peng,[6] Zhen Li,[3,7] Claudia Dastmalchi,[1] Gabriel Santpere,[3,8] André M.M. Sousa,[3] Andrew T.N. Tebbenkamp,[3] Navjot Kaur,[3] Forrest O. Gulden,[3] Michael S. Breen,[9,10,11,12] Lindsay Liang,[1] Michael C. Gilson,[1] Xuefang Zhao,[13,14,15] Shan Dong,[1] Lambertus Klei,[16] A. Ercument Cicek,[17,18] Joseph D. Buxbaum,[9,10,11,19] Homa Adle-Biassette,[20]

(Author list continued on next page)

[1]Department of Psychiatry, UCSF Weill Institute for Neurosciences, University of California, San Francisco, San Francisco, CA 94158, USA
[2]Laboratory of Genetics, University of Wisconsin-Madison, Madison, WI 53706, USA
[3]Department of Neuroscience and Kavli Institute for Neuroscience, Yale School of Medicine, New Haven, CT 06510, USA
[4]Department of Integrated Biomedical and Life Science, Korea University, Seoul 02841, Republic of Korea
[5]School of Biosystem and Biomedical Science, College of Health Science, Korea University, Seoul 02841, Republic of Korea
[6]Department of Statistics and Data Science, Carnegie Mellon University, Pittsburgh, PA 15213, USA
[7]Department of Neurosciences, University of California, San Diego, San Diego, CA 92093, USA
[8]Neurogenomics Group, Research Programme on Biomedical Informatics, Hospital del Mar Medical Research Institute, Department of Experimental and Health Sciences, Universitat Pompeu Fabra, 08003 Barcelona, Catalonia, Spain
[9]Seaver Autism Center for Research and Treatment, Icahn School of Medicine at Mount Sinai, New York, NY 10029, USA
[10]Department of Psychiatry, Icahn School of Medicine at Mount Sinai, New York, NY 10029, USA
[11]Mindich Child Health and Development Institute, Icahn School of Medicine at Mount Sinai, New York, NY 10029, USA
[12]Department of Genetics and Genomic Sciences, Icahn School of Medicine at Mount Sinai, New York, NY 10029, USA
[13]Center for Genomic Medicine and Department of Neurology, Massachusetts General Hospital, Boston, MA 02114, USA
[14]Department of Neurology, Harvard Medical School, Boston, MA 02115, USA
[15]Program in Medical and Population Genetics and Stanley Center for Psychiatric Research, Broad Institute, Cambridge, MA 02142, USA
[16]Department of Psychiatry, University of Pittsburgh School of Medicine, Pittsburgh, PA 15213, USA
[17]Department of Computer Engineering, Bilkent University, Ankara 06800, Turkey
[18]Computational Biology Department, Carnegie Mellon University, Pittsburgh, PA 15213, USA
[19]Friedman Brain Institute, Icahn School of Medicine at Mount Sinai, New York, NY 10029, USA
[20]Department of Pathology, Lariboisière Hospital, APHP, Biobank BB-0033-00064, and Université de Paris, 75006 Paris, France
[21]Department of Neurology, Yale University School of Medicine, New Haven, CT 06511, USA
[22]UMRS1127, Sorbonne Université, Institut du Cerveau et de la Moelle Épinière, 75013 Paris, France
[23]Center for Integrative Brain Research, Seattle Children's Research Institute, Seattle, WA 98101, USA
[24]Brotman Baty Institute for Precision Medicine, Seattle, WA 98195, USA
[25]Department of Pediatrics, University of Washington, Seattle, WA 98105, USA

(Affiliations continued on next page)

SUMMARY

Gene expression levels vary across developmental stage, cell type, and region in the brain. Genomic variants also contribute to the variation in expression, and some neuropsychiatric disorder loci may exert their effects through this mechanism. To investigate these relationships, we present BrainVar, a unique resource of paired whole-genome and bulk tissue RNA sequencing from the dorsolateral prefrontal cortex of 176 individuals across prenatal and postnatal development. Here we identify common variants that alter gene expression (expression quantitative trait loci [eQTLs]) constantly across development or predominantly during prenatal or postnatal stages. Both "constant" and "temporal-predominant" eQTLs are enriched for loci associated with neuropsychiatric traits and disorders and colocalize with specific variants. Expression levels of more than 12,000 genes rise or fall in a concerted late-fetal transition, with the transitional genes enriched for cell-type-specific genes and neuropsychiatric risk loci, underscoring the importance of cataloging developmental trajectories in understanding cortical physiology and pathology.

INTRODUCTION

The human nervous system develops slowly over several decades, starting during embryogenesis and extending postnatally through infancy, childhood, adolescence, and young adulthood (Keshavan et al., 2014; Shaw et al., 2010; Silbereis et al., 2016; Tau and Peterson, 2010). Over this time, myriads of functionally distinct cell types, circuits, and regions are formed (Hu et al.,

This is an open access article under the CC BY license (http://creativecommons.org/licenses/by/4.0/).

OPEN
ACCESS
CellPress

Jean-Leon Thomas,[21,22] Kimberly A. Aldinger,[23,24] Diana R. O'Day,[25] Ian A. Glass,[25] Noah A. Zaitlen,[26]
Michael E. Talkowski,[13,14,15] Kathryn Roeder,[6,18] Matthew W. State,[1,27] Bernie Devlin,[16] Stephan J. Sanders,[1,27,33,*] and
Nenad Sestan[3,28,29,30,31,*]

[26]Department of Medicine, University of California, San Francisco, San Francisco, CA 94158, USA
[27]Institute for Human Genetics, University of California, San Francisco, San Francisco, CA 94158, USA
[28]Department of Psychiatry, Yale University School of Medicine, New Haven, CT 06520, USA
[29]Department of Genetics, Yale University School of Medicine, New Haven, CT 06520, USA
[30]Department of Comparative Medicine, Program in Integrative Cell Signaling and Neurobiology of Metabolism, Yale School of Medicine, New Haven, CT 06510, USA
[31]Program in Cellular Neuroscience, Neurodegeneration, and Repair and Yale Child Study Center, Yale School of Medicine, New Haven, CT 06510, USA
[32]These authors contributed equally to this work
[33]Lead Contact
*Correspondence: stephan.sanders@ucsf.edu (S.J.S.), nenad.sestan@yale.edu (N.S.)
https://doi.org/10.1016/j.celrep.2020.03.053

2014; Lui et al., 2011; Silbereis et al., 2016). To produce distinct structures and circuits, neural cells are born in an immature state and undergo a variety of molecular and morphological changes as they differentiate, migrate, and establish circuits. Consequently, the characteristics of a given cell and brain region at a given time offer only a snapshot of organogenesis and brain function, necessitating consistent profiling across development.

The molecular and cellular processes underlying development of the nervous system rely on the diversity of transcripts and their precise spatiotemporal regulation (Bae and Walsh, 2013; Silbereis et al., 2016). Functional genomic analyses of the developing human brain have revealed highly dynamic gene expression and epigenetic changes during prenatal and early postnatal development (Kang et al., 2011; Li et al., 2018) versus comparative stability over several decades of adulthood (Colantuoni et al., 2011; Jaffe et al., 2018; Kang et al., 2011; Li et al., 2018; Pletikos et al., 2014). Disruption of developmentally dynamic regulatory processes is likely to contribute to neurodevelopmental and neuropsychiatric disorders (Birnbaum and Weinberger, 2017; Breen et al., 2016; Geschwind and Flint, 2015; McCarroll and Hyman, 2013; Rosti et al., 2014; Sestan and State, 2018; Turner and Eichler, 2019). In keeping with this expectation, spatiotemporal expression patterns have implicated mid-fetal brain development as a vulnerable process and the prefrontal cortex as a vulnerable region for autism spectrum disorder (ASD) and schizophrenia risk genes (Chang et al., 2015b; Gulsuner et al., 2013; Li et al., 2018; Network and Pathway Analysis Subgroup of the Psychiatric Genomics Consortium, 2015; Parikshak et al., 2013; Satterstrom et al., 2020; Willsey et al., 2013; Xu et al., 2014). More generally, atypical trajectories of brain maturation have been described in ASD, schizophrenia, and other neuropsychiatric traits and disorders (Birnbaum and Weinberger, 2017; Courchesne et al., 2007; Ecker et al., 2015; Insel, 2010; Keshavan et al., 2014; Shaw et al., 2010; Tang and Gur, 2018). Given that neuropsychiatric disorders have discrete ages of onset and progression and may arise because of genetic or environmental insults at various times during the life of an individual, there is a clear need to examine gene expression and neuropsychiatric risk across the span of human brain development.

In addition to spatiotemporal variation, genetic sequence variants also affect gene expression levels, which can contribute to differences in brain structure, function, and behavior (Elliott et al., 2018). Several laboratories and consortia have systematically identified such expression quantitative trait loci (eQTLs) in numerous tissues, including the brain (Akbarian et al., 2015; Dobbyn et al., 2018; Fromer et al., 2016; Gibbs et al., 2010, GTEx Consortium, 2015; Heinzen et al., 2008; Jaffe et al., 2018; Liu et al., 2010; Myers et al., 2007; Wang et al., 2018; BrainSeq: A Human Brain Genomics Consortium, 2015), but fewer include the developing human brain (Colantuoni et al., 2011; Jaffe et al., 2018; Kang et al., 2011; O'Brien et al., 2018; Walker et al., 2019). Therefore, developmentally regulated eQTLs are sparsely represented in the current catalog of human brain eQTLs, highlighting the need for additional resources. Such eQTL catalogs offer the potential to gain insight into the functional consequences of the hundreds of coding and noncoding genetic loci that have been associated with neuropsychiatric traits and disorders, including developmental delay, ASD, educational attainment, schizophrenia, major depressive disorder, and Alzheimer's disease (Deciphering Developmental Disorders Study, 2017, Schizophrenia Working Group of the Psychiatric Genomics Consortium, 2014; Grove et al., 2019; Kosmicki et al., 2016; Lee et al., 2018; Sanders et al., 2015, 2017; Satterstrom et al., 2020).

To help fill this gap, we generated BrainVar, a unique resource of whole-genome sequencing (WGS) paired with bulk tissue RNA sequencing (RNA-seq) of 176 samples from the human dorsolateral prefrontal cortex (DLPFC) across development, from 6 post-conception weeks to young adulthood (20 years). We focused our analyses on the DLPFC because of its importance in higher-order cognition (Silbereis et al., 2016) and the observation that many risk genes for ASD and schizophrenia are co-expressed in the DLPFC during mid-fetal development (Gulsuner et al., 2013; Li et al., 2018; Network and Pathway Analysis Subgroup of the Psychiatric Genomics Consortium, 2015; Parikshak et al., 2013; Willsey et al., 2013). We present a systematic description of this resource, including demographics, gene expression across development, gene co-expression modules, and eQTLs. We describe interactions between these factors and comparisons with the BrainSpan dataset, cell-type-specific genes, and loci associated with neuropsychiatric traits and disorders. Our analysis replicates the late-fetal transition observation, a dramatic shift in gene expression between mid-fetal

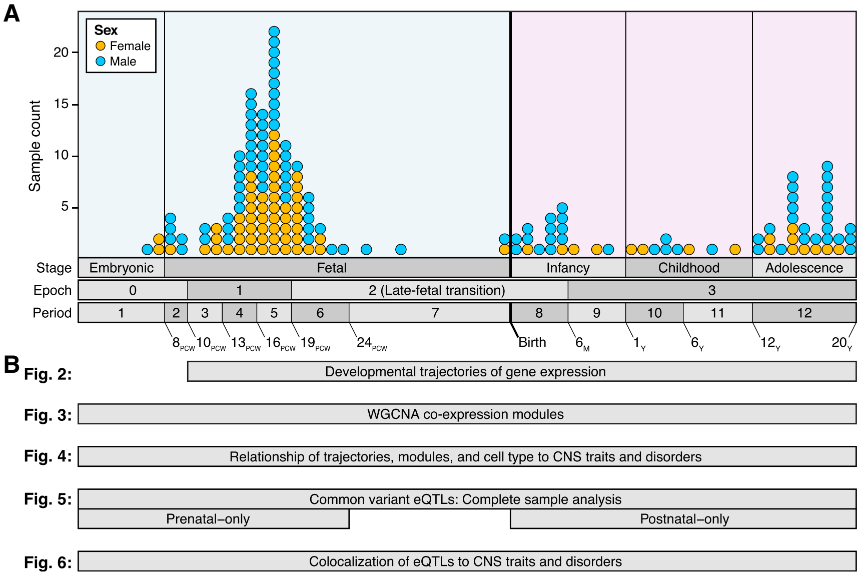

Figure 1. Overview of the Dataset and the Analysis

(A) 176 samples from the dorsolateral prefrontal cortex (DLPFC) of the developing human brain were processed to generate RNA-seq gene expression data and WGS data (top). The distribution of the samples is shown by sex (color) and developmental stage (x axis). Periods were defined previously (Kang et al., 2011), and epochs are defined as a superset of periods based on principal component analysis of these RNA-seq data (Figure 2).

(B) Analyses conducted using these data. The width of each box corresponds to the samples included in each analysis.

See also Table S1 and Figure S1.

development and infancy (Li et al., 2018); refines the timing of this event; and delineates the degree to which each gene is involved. We also identify 252,629 *cis*-eQTLs affecting 8,421 genes and classify their effects as prenatal-predominant, postnatal-predominant, or constant across brain development. Finally, we identify eQTLs that co-localize with genome-wide association study (GWAS) loci, linking specific genes to neuropsychiatric phenotypes.

RESULTS

Description of the Cohort and Data Generation

To characterize gene expression across prenatal and postnatal development of the human DLPFC and to identify genetic variants associated with expression changes, post mortem tissue was obtained from 176 de-identified, clinically unremarkable donors (genotypic sex: 104 male, 72 female) without known neuropsychiatric disorders or large-scale genomic abnormalities, ranging between 6 post-conception weeks and 20 years of age (Figure 1; Table S1). In keeping with prior analyses (Kang et al., 2011), we assign these samples to 12 developmental periods, which we group into four developmental epochs (Figures 1 and 2). Gene expression data were generated using RNA-seq from tissue dissected from the DLPFC (corresponding mainly to Brodmann area 46) or from the frontal cerebral wall (donors younger than 10 post-conception weeks). WGS data (31.5× median coverage) were generated simultaneously from DNA isolated from the same individuals.

Data Processing

RNA-seq reads were aligned and converted to log base 2 counts per million (log_2CPM) per gene (STAR Methods), with 23,782 genes meeting minimum expression criteria. We restricted further analysis to these 23,782 cortically expressed genes, of which 16,296 (68.5%) encode proteins, whereas 7,486 (31.5%) are noncoding, including long noncoding RNA (lncRNA) (12.6% of total) and antisense (9.2% of total) genes (Table S2). For the 14 samples also profiled in BrainSpan (Li et al., 2018), gene expression was highly correlated per sample and per gene (Figure S1). In both datasets, the first principal component of gene expression is strongly correlated with developmental age (Figures 2A and S1). All samples were genotypically concordant between the WGS and RNA-seq data (Regier et al., 2018). Ancestry correlated strongly between principal-component analysis clusters and self-report (STAR Methods).

Temporal Dynamics of Gene Expression

Prior analysis of the 40 brains in the BrainSpan cohort identified developmental age as the greatest source of between-sample variance in gene expression, especially during a "late-fetal transition" between 22 post-conception weeks and 6 postnatal months (Kang et al., 2011; Li et al., 2018). We replicate these findings in BrainVar. The first principal component explains 42% of the variance in gene expression and is highly correlated with developmental age (partial R^2 = 0.88; Figures 2A and S2; similar results when excluding the 14 overlapping samples), with the greatest changes occurring in late fetal development and early infancy (Figure 2A).

Using the increased resolution from the 176 brains in BrainVar, we show that the late-fetal transition begins around 19 post-conception weeks (start of period 6) and that the most dramatic changes are complete by 6 postnatal months (end of period 8); we label this transitional phase as epoch 2 (Figure 2A). Considering the nine samples younger than 10 post-conception weeks (periods 1–2), we also observe an "early-fetal transition," i.e., a coordinated shift in embryonic and early fetal development, which we label epoch 0 (Figure 2A).

To identify the specific genes that change in the late-fetal transition, we performed a trajectory analysis on the 167 samples in epochs 1–3; we excluded epoch 0 because of the sparse sampling before and during the early-fetal transition. Remarkably, over half of the genes expressed in the cortex exhibit a persistent, progressive, and statistically significant expression variance across this late-fetal transition (Figure 2B). We identified three distinct trajectories, with 6,934 "rising" genes (higher

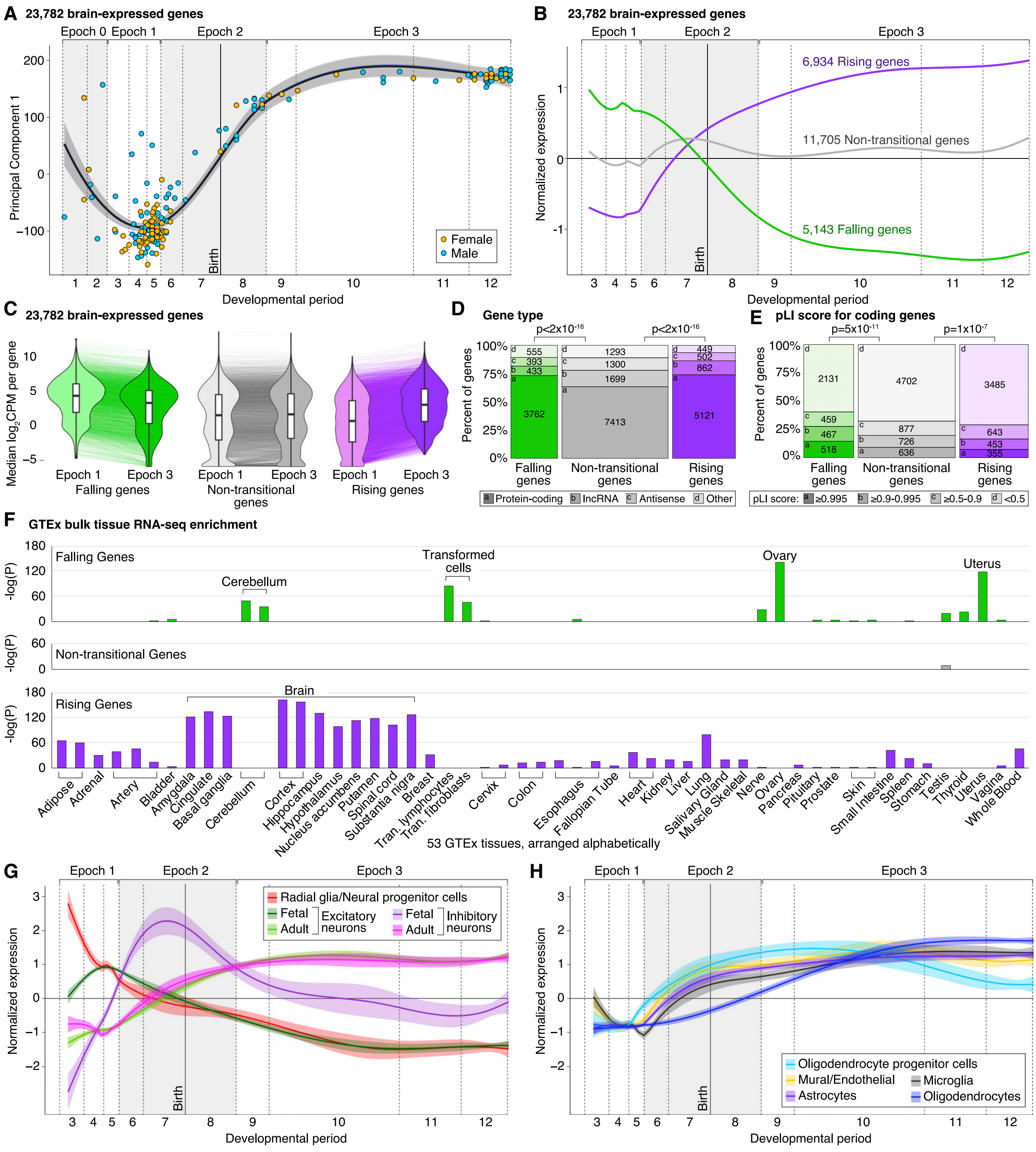

Figure 2. Temporal Trajectories of Gene Expression in the Human DLPFC

(A) Gene expression log base 2 counts per million (log_2CPM) for each sample was used to calculate principal components (Figure S2). The first principal component (PC1) explains 42% of the variance between samples, and 81% of variance in PC1 is explained by developmental stage (Figure S2). The changes in PC1 over time were used to define four "epochs" of gene expression. Dotted lines represent the boundaries of the indicated developmental period as defined previously (Kang et al., 2011).

(B and C) Trajectory analysis identifies three sets of genes with similar developmental profiles across the late-fetal transition in epoch 2 (B; Table S2). For each group, the expression over time, normalized by the interquartile range and locally estimated scatterplot smoothing (LOESS), is shown as a line, with the narrow

(legend continued on next page)

postnatal expression), 5,143 "falling" genes (higher prenatal expression), and 11,705 "non-transitional" genes (no statistically significant change). Considering more than three trajectories further split gene sets by variance rather than developmental profile (STAR Methods). Similar trajectories are observed for these three gene lists in the BrainSpan DLPFC data (Figure S2). Emphasizing the magnitude of this transition, the first principal component of the 11,705 non-transitional genes explains only 18.4% of the variance in gene expression and is weakly correlated with developmental age (partial $R^2 = 0.3$; Figure S2).

The magnitude of the changes in individual genes' expression levels across late-fetal transition can be estimated by calculating the difference in $\log_2$CPM expression between epoch 3 and epoch 1, ranging from 12.1 (*OPALIN*, a component of myelin) to -9.2 (*IGF2BP1*, an IGF2 binding protein); for context, the median epoch 3-to-epoch 1 changes in $\log_2$CPM values were 2.1, 0.1, and -1.1 for rising, non-transitional, and falling, respectively. The majority of changes in gene expression reflected relative amplification or attenuation of expression levels rather than binary presence/absence of expression, with only 621 rising genes and 95 falling genes specific to epoch 3 or 1 (defined as $\log_2$CPM ≤ -5 in the other epoch; Figure 2C; Table S2).

Characteristics of Transitional and Non-transitional Genes

Compared with rising and non-transitional genes, falling genes had the highest median expression in epoch 1 ($p < 2 \times 10^{-16}$) and epoch 3 ($p < 2 \times 10^{-16}$, Wilcoxon rank-sum test [WRST]; Figure 2C) and the highest fraction of protein-coding genes ($p < 2 \times 10^{-16}$, Fisher's exact test [FET]; Figure 2D) and were highly enriched for genes with high probability loss-of-function intolerant (pLI) scores ($p = 5 \times 10^{-11}$, WRST; Figure 2E). High pLI scores reflect detection of fewer protein-truncating variants than expected (Lek et al., 2016), suggesting that loss-of-function mutations in the gene are disfavored by natural selection (i.e., the gene is haploinsufficient). Rising genes had a similar proportion of protein-coding genes as falling genes ($p = 0.94$, FET; Figure 2D) but were depleted for genes with high pLI scores ($p = 1 \times 10^{-7}$, WRST; Figure 2E). If the timing of a gene's highest expression corresponds to the timing of its most critical functions, then the pLI difference between falling and rising genes suggests that prenatal development is especially sensitive to haploinsufficiency.

Compared with RNA-seq data from 53 adult tissues (GTEx Consortium, 2015), falling genes were only enriched in non-cortical tissues (driven by genes related to RNA transcription and cell division; Table S2), whereas rising genes were enriched for many brain regions, including the adult cortex and excluding cerebellum (Figure 2F), highlighting the distinctions between the fetal and adult cortex. Non-transitional genes had the lowest proportion of protein-coding genes and were expressed ubiquitously across adult tissues (Figure 2).

Cell Type Dynamics across Development

To capture the contribution of changing cell type proportions to gene expression profiles, we assessed expression trajectories of genes specific to each of ten cortical cell types from prenatal (Nowakowski et al., 2017) and postnatal human brain (Li et al., 2018; Velmeshev et al., 2019). The estimated profiles of all ten cell types vary dramatically across epoch 2, with radial glia/neural progenitor cells and fetal neurons decreasing as mature neurons and other glial cells increase (Figures 2G and 2H); this pattern is replicated in BrainSpan DLPFC samples (Figure S2). These analyses support the hypothesis that varying cell type proportions are major contributors to the late-fetal transition in the DLPFC (Li et al., 2018), but distinguishing cellular composition effects from differential expression within a cell type will require single-cell data from across this age range.

Co-expression Modules in the Developing Human Cortex

To further characterize the relationships between the 23,782 cortically expressed genes, we applied a weighted gene co-expression network analysis (WGCNA) (Langfelder and Horvath, 2008) to define 19 consensus modules that included 10,459 genes (Figures 3A and S3; Table S3). As expected, genes within each module shared functional roles (Figure 3B), temporal trajectories of gene expression (Figures 3C and 3D), regulatory transcription factors (Figure S3), and cell type enrichment (Figures 3E and 3F). Module preservation analysis using BrainSpan data (Li et al., 2018) identified similar co-expression patterns across brain regions, especially independent DLPFC samples (Figure 3G).

Similar to transitional genes (Figure 2), multidimensional scaling of the module eigengenes demonstrated that developmental age accounted for 44.7% and 36.1% of the variance in the first two dimensions. Considering the position of the 19 modules along these two dimensions and the developmental trajectories of the genes in each module, we identified five groups of related modules (Figures 3A and 3B). Group 1 modules (M1 black, M2 royal blue, M3 green-yellow, and M4 yellow) are enriched for falling genes, whereas group 5 modules (M16 blue, M17 silver, M18 light cyan, and M19 turquoise) are enriched for rising genes (Figure 3D). The remaining three groups (2, 3, and 4) are enriched for non-transitional genes (Figure 3D).

Five modules are of particular note. The M2 royal blue module (group 1) captures cell cycle ontology and is enriched in

95% CI in gray. These three groups are further characterized by plotting (C) the median $\log_2$CPM across all samples in epoch 1 and epoch 3, with the difference for each gene shown as a line.

(D) The relative proportion of Gencode protein-coding and noncoding genes with gene counts.

(E) The distribution of probability loss-of-function intolerance (pLI) scores for protein-coding genes (Lek et al., 2016) with gene counts.

(F) Enrichment in the most tissue-specific genes from the 53 tissues with bulk tissue RNA-seq data from the Genotype-Tissue Expression Consortium (GTEx) (GTEx Consortium, 2015).

(G) Pattern of expression for ten cell type-specific genes (Table S2) for each of five neuronal lineage cell types (LOESS with 95% CI).

(H) Analysis in (G) repeated for five glial lineage cell types.

OPC, oligodendrocyte progenitor cell. Statistical analyses: (A) principal component analysis; (B) longitudinal mixture model with Gaussian noise; (D) FET; (E) two-sided WRST; (F) t-test. See also Table S2 and Figures S1 and S2.

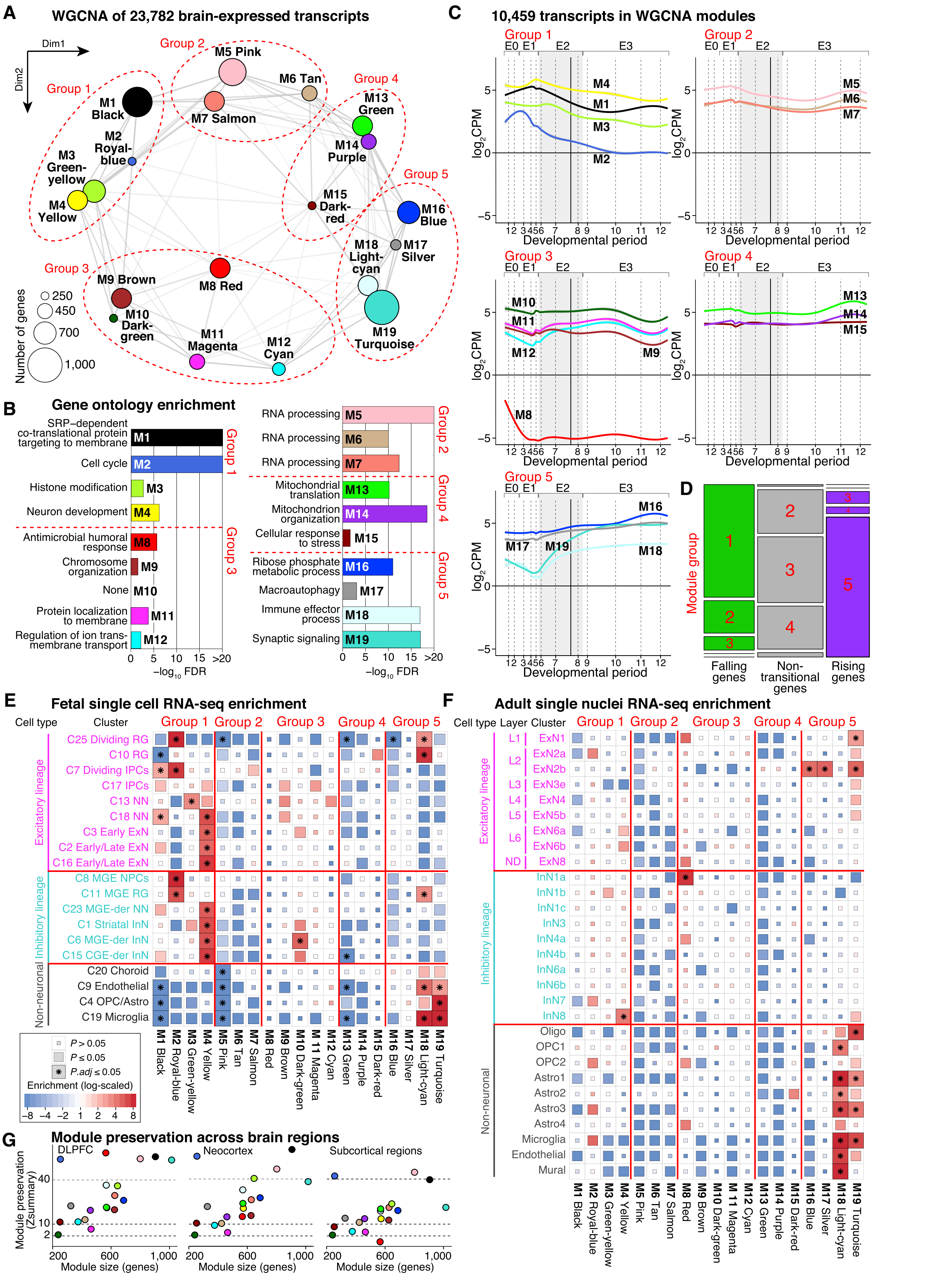

(legend on next page)

neuroprogenitor cells, radial glia, and intermediate progenitor cells. The M4 yellow module (group 1) is enriched for numerous ontology terms related to neuronal development, contains genes specific to neuronal stem cells (e.g., *NCAM1/ncam* and *PROM1/cd133*), and is highly enriched for genes related to maturing excitatory and inhibitory neurons. The M8 red module (group 3) is enriched for ontology terms relating to cell fate and morphogenesis, is highly enriched for noncoding genes, and has an expression peak in early fetal development, capturing many genes that are involved in early-fetal transition (Figure 2A). Several genes associated with regional patterning in non-cortical tissues, including the hindbrain (e.g., *UNCX* and *CCDC140*) and hypothalamus (e.g., *DMBX1* and *SOX14*), are expressed at high levels in this module. The M18 light cyan and M19 turquoise modules (group 5) are strongly enriched in glial and other non-neuronal cell clusters; accordingly, both modules are enriched for ontology terms related to immune responses. The M19 turquoise module is also enriched in excitatory neurons in the post-natal cortex and ontology terms relating to synaptic signaling and neurotransmitter transport.

Intersection of Developmental Expression with Human Traits and Disorders

We next considered the intersection between genes associated with developmental trajectories, modules, or cell types and genes associated with ten human traits and disorders. For ASD and developmental delay with and without seizures, we used gene lists derived from exome association studies of rare and *de novo* variants (Deciphering Developmental Disorders Study, 2017; Heyne et al., 2018; Satterstrom et al., 2020). For educational attainment, attention deficit hyperactivity disorder (ADHD), schizophrenia, major depressive disorder, multiple sclerosis, Parkinson's disease, and Alzheimer's disease, we used genes within 10 kb of the lead SNP detected in GWASs (Chang et al., 2017; Demontis et al., 2019; Beecham et al., 2013; Lambert et al., 2013; Lee et al., 2018; Schizophrenia Working Group of the Psychiatric Genomics Consortium, 2014; Wray et al., 2018). For our analyses, we excluded genes within the major histocompatibility complex on chromosome 6 because of the complicated nature of this region (Schizophrenia Working Group of the Psychiatric Genomics Consortium, 2014).

Developmental delay, ASD, and educational attainment genes were enriched for falling genes ($p = 8.5 \times 10^{-6}$, $p = 5.1 \times 10^{-3}$, and $p = 2.4 \times 10^{-4}$ respectively; FET adjusted for 30 comparisons), consistent with a prenatal origin for aspects of their neurobiology. A non-significant trend toward enrichment for rising genes was observed for Parkinson's disease and Alzheimer's disease (Figure 4A; Table S4). The M4 yellow module was enriched for ASD and educational attainment genes, including *NRXN1*, *TCF4*, and *BCL11A* (Figure 4B; Table S4), and the M9 brown module (enriched for chromatin organization Gene Ontology terms and non-transitional genes; Figure 3) was enriched for genes associated with developmental delay and educational attainment, including *CDK13*, *PACS1*, and *EP300* (Figure 4B; Table S4).

Across the ten CNS traits and disorders, five cell type clusters (C) (Li et al., 2018; Nowakowski et al., 2017) showed significant enrichment in fetal brain (Figure 4C; Table S4) and none in adult brain (Figure S4; Table S4). ASD genes were enriched for C18 excitatory newborn neurons and C1 striatal interneurons (Figure 4C), in keeping with a role of excitatory and inhibitory lineages (Satterstrom et al., 2020). Both lineages were also enriched in educational attainment, specifically C3 early excitatory neurons and C6 medial ganglionic eminence (MGE)-derived interneurons, whereas genes associated with developmental delay with seizures were enriched in C15 caudal ganglionic eminence (CGE)-derived interneurons (Figure 4C). We observed a nominally significant trend toward enrichment of C19 microglia genes in multiple sclerosis and Alzheimer's disease.

Common Genetic Variants Regulating Gene Expression

We identified 6,573,196 high-quality SNPs and insertions or deletions (indels) from the WGS data using methods described previously (Werling et al., 2018), with an allele frequency of at least 5% in our prenatal (periods 1–6, n = 112) and postnatal (periods 8–12, n = 60) samples (Figure 1). To identify eQTLs within 1 Mb of a gene (eGene), we used linear regression for adjusted expression level (STAR Methods), with developmental period, sex,

Figure 3. Co-expression Modules in the Developing Human Cortex

(A) Weighted genome co-expression network analysis (WGCNA) identified 19 modules comprised of 10,459 of 23,782 expressed genes. Modules are shown as colored nodes plotted based on the first two dimensions from multidimensional scaling. The weight of the connecting lines (edges) represents the degree of correlation between module eigengenes.

(B) LOESS expression values across development are shown with 95% CIs for the 19 modules arranged in five groups based on proximity in (A) and similar temporal trajectories.

(C) Gene Ontology enrichment analysis for each module, showing only biological processes with the lowest false discovery rate (FDR).

(D) Mosaic plot showing the relationship between the five groups of co-expression modules (from A) and genes with falling, rising, or non-transitional temporal trajectories (Figure 2). The area is proportional to the number of genes in each bin. Detailed relationships between modules and temporal trajectories are shown in Figure S3.

(E) Enrichment between the 19 modules and the 200 genes most specific to 19 cell type clusters defined by single-cell RNA-seq data in the developing human cortex (Nowakowski et al., 2017).

(F) Enrichment between the 19 modules and the 200 genes most specific to 29 cell type clusters defined by single nucleus RNA-seq data in the adult human DLPFC (Li et al., 2018).

(G) Module preservation in independent BrainSpan samples (Li et al., 2018) from the same brain region (left), other cortical regions (center), and five subcortical regions (right).

SRP, signal recognition particle; C, cluster of single nuclei; L, cortical layer; ND, layer not defined; RG, radial glia; IPC, intermediate progenitor cell; NN, newborn neuron; ExN, excitatory neuron; InN, inhibitory neuron; CGE, caudal ganglionic eminence; MGE, medial ganglionic eminence. Statistical analysis: (A) WGCNA with consensus module detection from 100 random resamplings; (C) FET, corrected for gProfiler Gene Ontology pathways (10–2,000 term size); (E) FET corrected for 361 comparisons; (F) FET corrected for 551 comparisons; (G) FET corrected for 19 comparisons. See also Tables S2 and S3 and Figure S3.

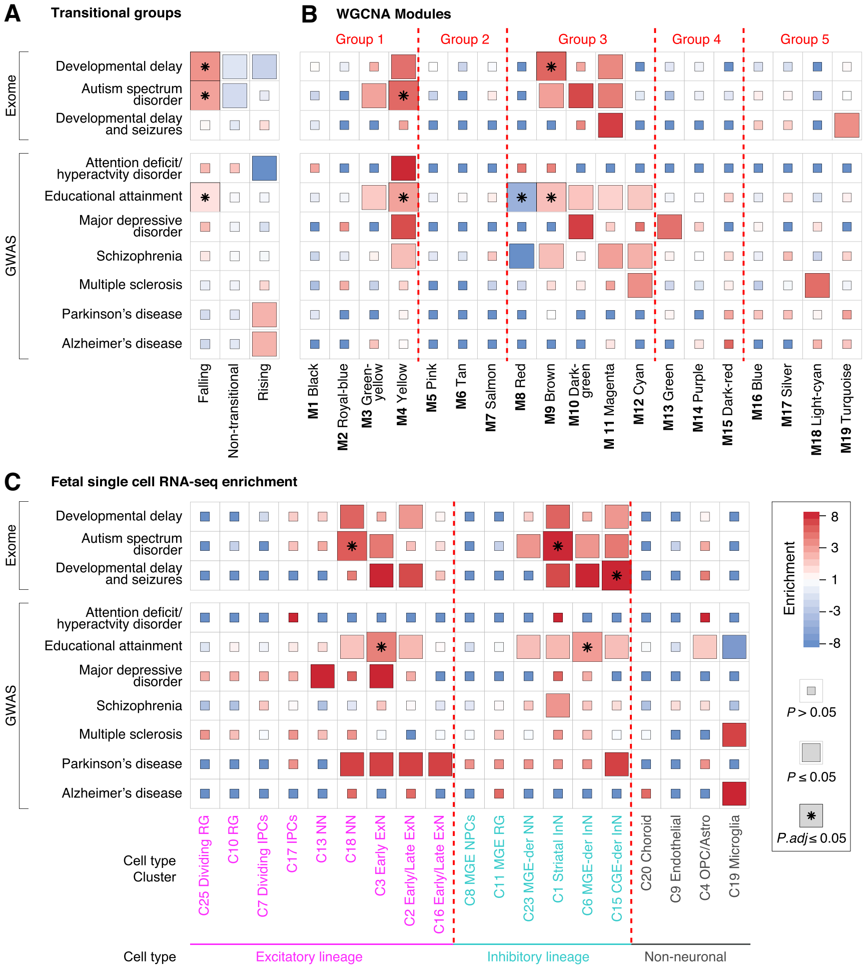

Figure 4. Expression of Genes Associated with CNS Traits and Disorders

(A) Genes from genome-wide significant loci were collated for ten CNS traits and disorders from exome sequencing or genome-wide association studies (GWASs). The enrichment is shown for the three trajectory groups (Figure 2).

(B) The analysis in (A) repeated for co-expression modules.

(C) The analysis in (A) repeated for genes enriched for cell type clusters from single-cell RNA-seq of the prenatal human brain.

Statistical analysis: (A) FET corrected for 30 comparisons; (B) FET corrected for 190 comparisons; (C) FET corrected for 190 comparisons. See also Tables S2 and S4, and Figure S4.

and the first five principal components for ancestry as covariates. Results were corrected for multiple comparisons using Benjamini-Hochberg (false discovery rate [FDR] $\leq$ 0.05). To distinguish temporal-predominant eQTLs, we performed three *cis*-eQTL analyses: all 176 samples (complete sample, 216,026 eQTLs of 5,728 eGenes), 112 prenatal samples (periods 1–6, 154,440 eQTLs of 4,378 eGenes), and 60 postnatal samples (periods 8–12, 51,528 eQTLs of 2,199 eGenes). These discovery rates are in line with similarly sized cohorts (Figure S5). The union of these three analyses identified 252,629 eQTLs of 8,421 eGenes (Table S5). As expected, the eQTLs are enriched for markers of active transcription derived from the human brain (Figure S5; Li et al., 2018; Reilly et al., 2015; Kundaje et al., 2015). We find that eQTL effect size and direction are correlated with prenatal whole brain (O'Brien et al., 2018) (Pearson's r = 0.73, $p \leq 1 \times 10^{-16}$; Figure 5A) and postnatal (adult) frontal cortex (Aguet et al., 2017) (Pearson's r = 0.73, $p \leq 1 \times 10^{-16}$; Figure 5A) from independent datasets.

Temporal Predominance of eQTLs

We leveraged the consistently processed prenatal and postnatal data in BrainVar to identify eQTLs with differing effect sizes across development (Figure 5B; STAR Methods). The majority of eQTLs were constant, reaching nominal significance in all three analyses with the same direction of effect (161,923 eQTLs, 64.1% of the total). Many eQTLs were prenatal-predominant, with significantly greater prenatal than postnatal effect sizes (24,760 eQTLs, 9.8%). Fewer eQTLs were postnatal-predominant, with significantly greater postnatal than prenatal effect sizes (9,352 eQTLs, 3.7%). The remaining 56,593 eQTLs (22.4%) showed a trend toward stronger prenatal effects (19.8%) or postnatal effects (2.6%). With larger sample sizes, we would expect a greater fraction of constant eQTLs to show some degree of temporal specificity, especially postnatal. Although the magnitude of effect varied across development for many eQTLs, we did not observe a single eQTL with opposing prenatal and postnatal directions of effect.

Temporal Predominance of eGenes

Most eGenes have more than one eQTL (5,538 of the 8,421, 65.8%). Defining the top eQTL per eGene as that with the lowest FDR-significant p value in any of the three sample sets (Table S5), we identified 2,977 (35.4% of total) constant eGenes, 1,691 (20.1%) prenatal-predominant eGenes, and 1,145 (13.6%) postnatal-predominant eGenes (Figure 5B). The remaining 2,608 eGenes (31.0%) trend toward prenatal (25.1%) or postnatal (5.9%) effects. Because of linkage disequilibrium (LD), *cis*-eQTLs for an eGene are likely to have a similar direction and magnitude of effect; accordingly, the temporal category of the top eQTL matched the majority of eQTLs for 88.2% of all eGenes (7,425 eGenes; Figure S5).

To validate the prenatal- and postnatal-predominant eQTLs, we evaluated their performance in independent datasets. In prenatal whole brain (O'Brien et al., 2018), we observed stronger correlation for the effects of prenatal-predominant (r = 0.54, $p = 3.9 \times 10^{-22}$) than postnatal-predominant eGenes (r = 0.36, $p = 1.1 \times 10^{-3}$; Figure 5C). In contrast, in postnatal frontal cortex

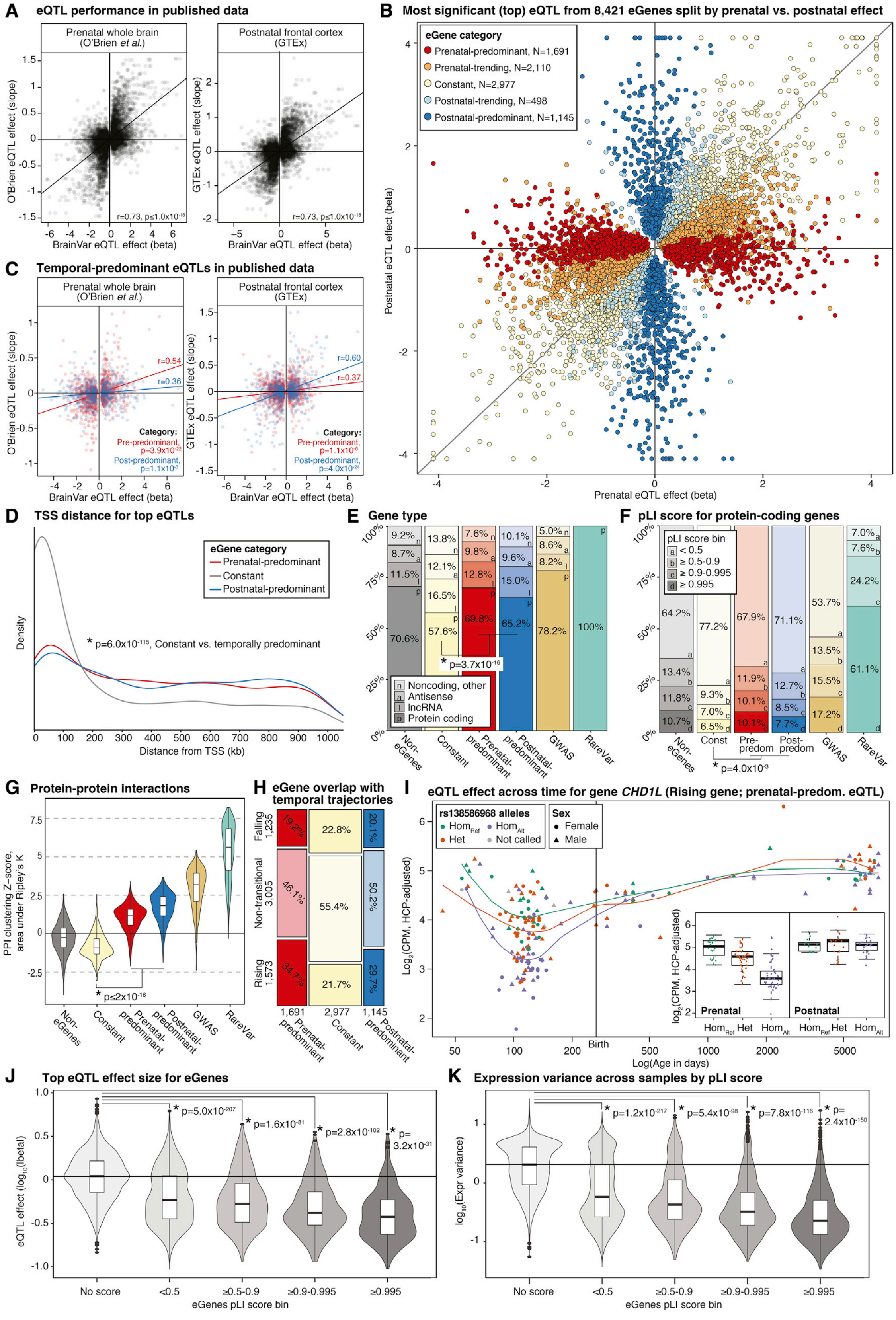

(legend on next page)

(Aguet et al., 2017), stronger correlations were observed for the effects of postnatal-predominant ($r = 0.60$, $p = 4.0 \times 10^{-24}$) than prenatal-predominant eGenes ($r = 0.37$, $p = 1.1 \times 10^{-6}$; Figure 5C).

Characteristics of Genes Influenced by eQTLs

The top eQTL for constant eGenes is closer to the transcription start site than that for temporal-predominant eGenes (median: 92,223 bp versus 403,702 bp, $p \leq 2 \times 10^{-16}$, two-sided WRST; Figure 5D). However, $-\log_{10}(P)$ values increased with proximity to the transcription start site for constant and temporal-predominant eQTLs (for both sets, $p \leq 2 \times 10^{-16}$, linear regression), as did eQTL effect size to a small degree (constant $p = 1.0 \times 10^{-12}$, temporal-predominant $p = 0.03$, linear regression). Compared with constant eGenes, temporal-predominant eGenes also included a higher proportion of protein-coding genes (odds ratio [OR] = 1.56, 95% confidence interval [CI]: 1.40–1.74), $p = 3.7 \times 10^{-16}$, two-sided FET; Figure 5E), genes with high pLI scores (pLI $\geq$ 0.995; OR = 1.45 [95% CI: 1.12–1.90], $p = 0.004$, two-sided FET; Figure 5F), and greater connectivity in protein-protein interaction (PPI) networks (median Z score 1.45 versus -0.91, $p \leq 2 \times 10^{-16}$, two-sided WRST; Figure 5H).

Given the dynamic expression profiles over development (Figure 2), we expected prenatal-predominant eGenes to be enriched for falling genes and postnatal-predominant eGenes to be enriched for rising genes. We did not observe these effects (Figure 5I). Instead, the prenatal-predominant eGenes are enriched for rising genes (OR = 1.5 [95% CI: 1.3–1.6], $p = 1.1 \times 10^{-10}$, two-sided FET; for example, Figure 5J), and pre- and postnatal-predominant eGenes are depleted for falling genes (prenatal OR = 0.79 [95% CI: 0.69–0.9], $p = 4.2 \times 10^{-4}$; postnatal OR = 0.85 [0.73–0.997], $p = 0.04$; two-sided FET). Instead, we observed coordination between the timing of eQTLs' strongest effects and the timing of eGenes' greatest expression variation between samples (STAR Methods). Prenatal-predominant eGenes are strongly enriched for genes with greater prenatal variance (OR = 2.3, $p = 4.0 \times 10^{-52}$, two-sided FET) and depleted for genes with greater postnatal variance (OR = 0.36, $p = 1.6 \times 10^{-15}$, two-sided FET). Postnatal-predominant eGenes show a complementary but weaker pattern of enrichment for genes with greater postnatal variance (OR = 1.1, $p = 0.25$, two-sided FET) and depletion of genes with greater prenatal variance (OR = 0.76, $p = 7.0 \times 10^{-5}$, two-sided FET). Considering the role of selective pressure, we observed that genes with higher pLI scores also had lower eQTL effect sizes ($p = 4.0 \times 10^{-36}$; two-sided WRST; Figure 5J) as well as lower expression variance between samples (Figure 5K).

eQTLs in Human Traits and Disorders

The differences in constant and temporal-predominant eGenes led us to consider how genes associated with neuropsychiatric traits and disorders (Figure 4) fit into this classification. Gene sets associated with traits by GWAS loci or exome sequence association followed the patterns of temporal-predominant eGenes but to a greater extent, with a higher proportion of protein-coding genes (Figure 5E), higher pLI scores (Figure 5F), and stronger clustering within PPI networks (Figure 5H).

At the variant level, we expect GWAS loci to be enriched for eQTLs in relevant tissues (Fromer et al., 2016; Nicolae et al., 2010). Using a permutation-based method accounting for LD structure, minor allele frequency (MAF), and gene density (STAR Methods), we tested four of the larger GWASs and observed eQTL enrichment for educational attainment, schizophrenia, and multiple sclerosis but not Alzheimer's disease (Figure S6; Table S6). We did not see evidence of the reverse hypothesis that eGenes are enriched for GWAS signals (Figure S6). Using a colocalization analysis, we looked for overlap between specific eQTL loci with educational attainment and schizophrenia GWAS loci using a posterior probability of colocalization threshold of 0.8. In the schizophrenia GWAS, 13 of 108 loci (12.0%) showed evidence of colocalization, including two prenatal-predominant and two postnatal-predominant eQTLs (Table S6). A lower proportion of educational attainment loci showed

Figure 5. Common Variant *cis*-eQTLs

(A) Effects of the top expression quantitative trait locus (eQTL) per gene regulated by an eQTL (eGene) with FDR $\leq$ 0.05 in the BrainVar analyses (x axis, union of results from complete sample, prenatal-only, and postnatal-only analyses) versus effects observed in the prenatal whole brain (O'Brien et al., 2018) (left) and postnatal frontal cortex (Aguet et al., 2017) (right, y axis).

(B) Prenatal (x axis) and postnatal (y axis) effects for the eQTLs with the smallest p value for 8,421 eGenes (points). The eQTLs are split into five categories based on temporal predominance using effect size and statistical thresholds; categories are represented by color.

(C) Effects of the top eQTL per eGene with FDR $\leq$ 0.05 from the prenatal-predominant (red) or postnatal-predominant (blue) eQTL categories from BrainVar (x axis) versus effects observed in the published datasets described in (A) (y axis).

(D) Density plot of the distance of top eQTLs per eGene from the transcription start site by eGene temporal category.

(E–G) Characteristics of non-eGenes, temporal-predominant eGenes, and disorder-associated genes are shown by plotting the (E) proportion of coding and noncoding genes, (F) proportion of genes with pLI scores in different bins, and (G) BioGRID protein-protein interactions (permuted Z scores from Ripley's K-net function; Cornish and Markowetz, 2014; the black line is the non-eGene median).

(H) Mosaic plot of the proportion of genes in each temporal trajectory with eGenes split by temporal category.

(I) Expression data binned by genotype for the top prenatal-predominant eQTL for *CHD1L*, a gene with a rising trajectory. Main panel: gene expression by sample age across development. Lines represent LOESS trajectories for expression in samples with each of three genotypes for rs138586968. Inset: boxplots for prenatal (left) and postnatal (right) samples with each of three rs138586968 genotypes.

(J) Distribution of eQTL effect size for eGenes binned by pLI scores. The black line represents the median of the transcripts with no pLI score.

(K) Distributions of between-sample variance in the expression level of expressed genes binned by pLI scores. The black line represents the median variance of the transcripts with no pLI score.

TSS, transcription start site; Statistical analysis: (A) and (C) Pearson correlation; (D) and (G), two-sided WRST test for constant versus other eGenes; (E) and (F), two-sided FET for constant versus other eGenes; (J) and (K), two-sided WRST test for each of four pLI bins versus genes with no pLI score. See also Tables S2 and S5 and Figure S5.

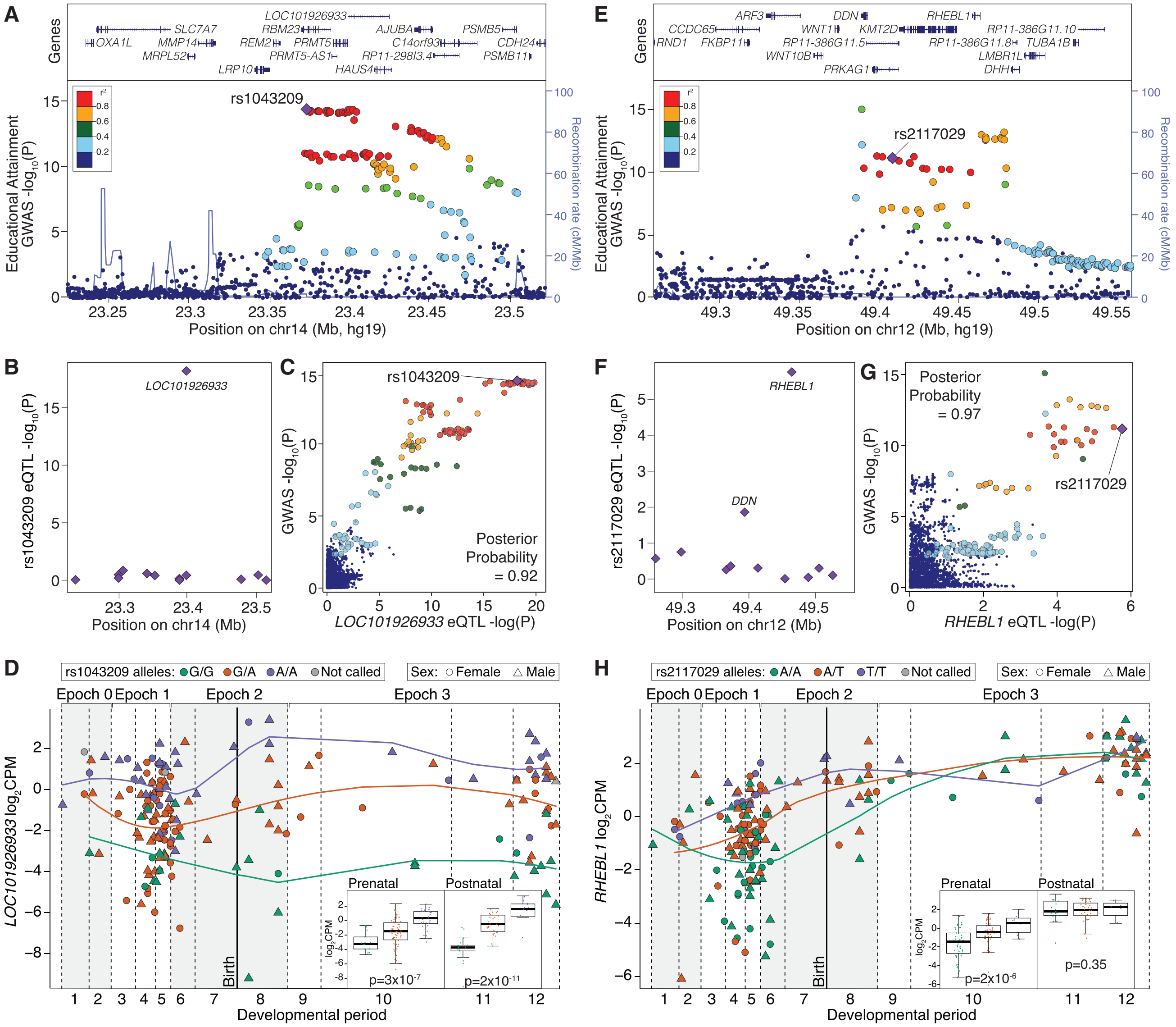

Figure 6. Colocalization of Two eQTLs with Educational Attainment GWAS Loci

(A) Statistical evidence of association with educational attainment for SNPs (points) alongside the recombination rate (blue line). Color shows the correlation with the SNP rs1043209 (Pruim et al., 2010).

(B) The statistical evidence for the SNP rs1043209 being an eQTL is shown for each gene within proximity of the locus. No other genes had high posterior probabilities for colocalization.

(C) The statistical evidence for being an eQTL for the noncoding RNA *LOC101926933* (x axis) is shown against the evidence for association with educational attainment (y axis) for each SNP (points).

(D) The expression of *LOC101926933* is shown for each sample across development with genotype at rs1043209, indicated by color.

(E–H) Another educational attainment locus that colocalized with the gene *RHEBL1* in the prenatal period only is shown, as in (A)–(D).

Statistical analysis: (B) colocalization analysis; (D) inset, ANOVA; (E) colocalization analysis; (H) inset, ANOVA. See also Tables S2 and S6 and Figure S6.

evidence of colocalization (4.1%, 52 of 1,271), including 14 prenatal-predominant and two postnatal-predominant eQTLs (Table S6). Focusing on multigenic loci with the strongest evidence of colocalization, we implicate specific genes and expression changes as the likely mechanism underlying the GWAS loci. SNPs associated with educational attainment at a chromosome 14 locus (Figure 6A) colocalized only with eQTLs for the lncRNA *LOC101926933* (also called *RP11-298I3.1*, *AL132780.1*, or *ENSG00000257285*; Figure 6B). Across the locus, the p values for the GWAS SNPs and *LOC101926933* eQTLs are highly correlated, resulting in a posterior probability for colocalization of 0.92 (Figure 6C); for this locus, eQTL effect size is similar across development (Figure 6D). We also observe colocalization of prenatal-predominant eQTLs with educational attainment or schizophrenia GWAS loci. For example, SNPs contributing to an educational attainment GWAS locus on chromosome 12

(Figure 6E) overlap specifically with eQTLs for the protein-coding gene *RHEBL1*, which encodes a brain-enriched G-protein activator of the mTOR pathway (Figure 6F). GWAS and *RHEBL1* eQTL prenatal p values are highly correlated and result in a posterior probability for colocalization of 0.97 (Figure 6G). We see a significantly greater eQTL effect size in prenatal compared with postnatal samples (p = 0.003; Figure 6H), with higher *RHEBL1* expression associated with increased educational attainment.

DISCUSSION

In this manuscript, we describe BrainVar, a unique resource of paired genome (WGS) and transcriptome (bulk tissue RNA-seq) data derived from 176 human DLPFC samples across prenatal and postnatal development (Figure 1). We identify 23,782 genes expressed during human cortical development, gene lists relating to developmental trajectories and co-expression, and common variants that alter gene expression (eQTLs). Our analyses show how these datasets relate to each other and to gene expression in cell types derived from single-cell RNA-seq data and to CNS traits and disorders derived from genomic analyses (exome sequencing and GWAS). In addition to developing a resource with utility for future studies of human development, neurobiology, and neuropsychiatric disorders, we also describe key biological insights, including the nature of the late-fetal transition in gene expression (Figures 2 and 3), an early-fetal transition (Figures 2 and 3), developmental processes and cell types implicated in CNS traits and disorders (Figure 4), eQTLs split by effect size across development (Figure 5), differing characteristics of genes with constant versus temporal-predominant eQTLs (Figure 5), and the application of this dataset to implicate specific genes at GWAS loci (Figure 6).

Principal component analysis identifies developmental age as the most important factor underlying the variance in gene expression in this dataset. The majority of this temporal variance occurs in two transitional phases (Figure 2), the early-fetal and late-fetal transitions. The early-fetal transition is a coordinated decrease in expression of multiple genes in early development (epoch 0; periods 1–2; 6–10 post-conception weeks) that coincides with the establishment of regional identity across the brain. Concordant with a possible role of the early-fetal transition in this process, the expression of several genes associated with non-cortical tissues (e.g., *UNCX* and *DMBX1*) is decreased during this period. In addition, we found that the early-fetal transition is captured in the M8 red module (Figure 3), which is enriched for lncRNA transcripts and Gene Ontology terms related to morphogenesis and cell fate.

The late-fetal transition between mid-fetal development and infancy involves over 12,000 genes with similar numbers rising and falling (Figure 2). Prior reports of humans (Li et al., 2018) and primates (Zhu et al., 2018) associated this transition with a reduction in intra- and inter-regional variation evident at the levels of bulk tissue and individual cell types. Our data similarly suggest that this transition represents a combination of changes in the relative proportions of various cell types and biological processes within these cells (Figures 2 and 3). Critically, the larger BrainVar sample set allowed us to define 19 post-conception weeks as the inflection point at which the late-fetal transition begins (Figure 2), further distinguishing the late-fetal transition from previously reported organotypic changes (Domazet-Lošo and Tautz, 2010; Kalinka et al., 2010; Li et al., 2018).

Although previous analyses have identified eQTLs in human brain tissue postnatally (Fromer et al., 2016; GTEx Consortium, 2015) and prenatally (Jaffe et al., 2018; O'Brien et al., 2018; Walker et al., 2019), no prior study has assessed the effect of genomic variation on gene expression across the whole of brain development, from embryogenesis through fetal development, infancy, childhood, and adolescence and into young adulthood. Consequently, we were able to identify temporal-predominant eQTLs that have a greater effect on expression prenatally or postnatally (Figure 5B). The eQTLs identified here were highly correlated with prior eQTL catalogs (Aguet et al., 2017; O'Brien et al., 2018) despite differing cohorts, methods, and analysis (Figures 5A and 5B). Furthermore, comparison with these independent catalogs support our temporal categorization of eQTLs, with prenatal-predominant eQTL effects more correlated in prenatal whole-brain and postnatal-predominant eQTL effects more correlated in the postnatal frontal cortex (Figure 5C).

Across multiple metrics, we observe dramatic differences between eGenes with constant and temporal-predominant eQTLs (Figures 5D–5G). Compared with other genes expressed in the cortex, genes affected by constant eQTLs are more likely to be noncoding and have low pLI scores and few protein-protein interactions. In contrast, genes regulated by eQTLs with a degree of temporal specificity are similar to genes for which we did not detect eQTLs. Critically, we find that pLI score, a measure of sensitivity to variation in genetic sequence, and eQTL effect size are inversely related (Figure 5J). Furthermore, prenatal-predominant eGenes are more common among rising genes, which have their highest expression during postnatal time. These observations suggest that developmental and evolutionary constraints limit the population frequency or effect of eQTLs on key developmental processes, a hypothesis that might be testable in future studies as additional information concerning spatiotemporal and cell type specificity of enhancers and eQTLs becomes available for a variety of tissues. Under this model, constant eQTLs with high effect sizes tend to influence genes that tolerate variation in expression (e.g., non-rate-limiting metabolic steps) or are non-critical to brain function, whereas temporal-predominant eQTLs tend to influence genes with critical roles that are sensitive to variation in genetic sequence but only to a small degree or at a stage in development when variation in expression of the gene is tolerated.

The eQTLs identified here also provide insights into CNS traits and disorders, with co-localization in 13 of 108 GWAS loci for schizophrenia and 52 of 1,271 GWAS loci for educational attainment, including the lncRNA *LOC101926933* and the protein-coding gene *RHEBL1* (Figure 6). *LOC101926933* remains largely uncharacterized, whereas *RHEBL1* (Ras homolog enriched in brain-like 1) is a highly conserved G-protein that activates mTOR (Bonneau and Parmar, 2012), a pathway that has been implicated previously in neurodevelopmental and neurodegenerative disorders (Lipton and Sahin, 2014). Our results suggest that higher expression of *RHEBL1*,

which may lead to greater mTOR activation, is associated with increased educational attainment. Of note, *RHEBL1* has a pLI score of 0, suggesting that loss of one allele does not lead to a selective disadvantage. Higher-resolution datasets across development, including single cells, additional brain regions, and larger sample sizes, along with complementary analyses of brains of individuals with neuropsychiatric disorders and rare genetic disorders, are likely to provide additional insights.

The combination of genomic and transcriptomic data across development allows us to interrogate human cortical development from a molecular perspective at a higher resolution than before. Understanding patterns of temporal and cell type specificity, along with eQTL colocalization to resolve GWAS loci, has already provided insights into the pathology underlying neuropsychiatric disorders. Further delineation of these patterns is likely to be critical for a detailed understanding of etiology as a foundation for therapeutic development.

STAR★METHODS

Detailed methods are provided in the online version of this paper and include the following:

- KEY RESOURCES TABLE
- LEAD CONTACT AND MATERIALS AVAILABILITY
- EXPERIMENTAL MODEL AND SUBJECT DETAILS
- METHOD DETAILS
  - Tissue dissection
  - RNA extraction and quality assessment
  - RNA-seq library preparation and sequencing
  - DNA extraction
  - Whole-genome sequencing
- QUANTIFICATION AND STATISTICAL ANALYSIS
  - WGS variant calling
  - RNA-seq alignment and gene-level read count quantification
  - RNA-seq normalization and technical artifact correction
- DATA QUALITY AND SAMPLE IDENTITY ASSESSMENT
  - Ancestry estimation
  - Estimation of biological and technical covariates in RNA-seq data
  - Comparison between BrainVar and BrainSpan
  - Transcriptome temporal trajectory estimation
  - Gene ontology functional enrichment for temporal trajectories
  - Assessing enrichment in tissue-specific genes from GTEx
  - Identifying genes enriched in cell types from single cell data
  - Enrichment of gene trajectories in temporal putative cis-regulatory elements
  - WGCNA network construction and module definition
  - WGCNA functional enrichment for module characterization
  - WGCNA module preservation
  - Clustering analysis in protein-protein interaction network
  - Cis-eQTL detection and classification
  - Alternative approaches for assigning eGenes to temporal categories
  - Assessment of ancestry differences in prenatal and postnatal sample sets using genomic control
  - Comparison with published eQTL studies
  - Distance between eQTLs and transcription start site
  - Overlap of eQTLs with H3K27ac
  - Enrichment of eQTLs in functional genomic elements
  - Test for differential expression variance in prenatal and postnatal stages
  - Gene sets associated with CNS traits and disorders
  - Enrichment of DLPFC eQTLs in SNPs associated with complex phenotypes
  - Gene-set analysis of eGenes and GWAS data
  - Co-localization analysis of CNS traits and disorders
- DATA AND CODE AVAILABILITY

SUPPLEMENTAL INFORMATION

Supplemental Information can be found online at https://doi.org/10.1016/j.celrep.2020.03.053.

ACKNOWLEDGMENTS

Data were generated as part of the PsychENCODE Consortium, supported by U01MH103339, U01MH103365, U01MH103392, U01MH103340, U01MH103346, R01MH105472, R01MH094714, R01MH105898, R21MH102791, R21MH105881, R21MH103877, and P50MH106934 awarded to Schahram Akbarian (Icahn School of Medicine at Mount Sinai), Gregory Crawford (Duke), Stella Dracheva (Icahn School of Medicine at Mount Sinai), Peggy Farnham (USC), Mark Gerstein (Yale), Daniel Geschwind (UCLA), Thomas M. Hyde (LIBD), Andrew Jaffe (LIBD), James A. Knowles (USC), Chunyu Liu (UIC), Dalila Pinto (Icahn School of Medicine at Mount Sinai), Nenad Sestan (Yale), Pamela Sklar (Icahn School of Medicine at Mount Sinai), Matthew State (UCSF), Patrick Sullivan (UNC), Flora Vaccarino (Yale), Sherman Weissman (Yale), Kevin White (UChicago), and Peter Zandi (JHU). This work was supported by funding provided by Autism Science Foundation postdoctoral fellowships (to D.W. and J.-Y.A.) and a research award (to S.J.S.); Simons Foundation Autism Research Initiative (SFARI) grants 574598 (to S.J.S.), 402281 (to S.J.S., M.W.S., B.D., and K.R.), and 573206 (to M.E.T.); National Institute for Mental Health (NIMH) grants R01 MH109901 (to S.J.S. and M.W.S.), R01 MH110928 (to S.J.S. and M.W.S.), U01 MH103339 (to M.W.S.), R01 MH111662 (to S.J.S. and M.W.S.), U01 MH105575 (to M.W.S.), U01 MH106874 (to N.S.), P50 MH106934 (to N.S.), R01 MH109904 (to N.S.), R01 MH110926 (to N.S.), U01 MH116488 (to N.S.), R37 MH057881 (to B.D.), and R01 MH115957 (to M.E.T.); National Institute of Child Health and Human Development (NICHD) grants R24 HD000836 (to I.A.G.), R01 HD081256 (to M.E.T.), and R01 HD096326 (to M.E.T.); National Heart, Lung, and Blood Institute (NHLBI) grant K25HL121295 (to N.A.Z.); National Human Genome Research Institute (NHGRI) grants U01HG009080 (to N.A.Z.) and R01HG006399 (to N.A.Z.); National Cancer Institute (NCI) grant R01CA227237 (to N.A.Z.); National Institute of Dental and Craniofacial Research (NIDCR) grant R03DE025665 (to N.A.Z.); United States Department of Defense (DoD) grant W81XWH-16-2-0018 (to N.A.Z.); and National Research Foundation of Korea grant 2019M3E5D3073568 (to J.-Y.A.). The project that gave rise to these results also received the support of a fellowship from "la Caixa" Foundation (ID 100010434 to G.S.). The fellowship code is LCF/BQ/PI19/11690010. We thank Thomas Lehner, Anjené Addington, Geetha Senthil, and Alexander Arguello at the NIMH for supporting the PsychENCODE Consortium, the Yale Center for Genome Analysis for generating the RNA-seq data, GENEWIZ for generating the WGS data, and Sentieon for use of a computationally efficient implementation of GATK Haplotype Caller.

AUTHOR CONTRIBUTIONS

Conceptualization, K.R., M.W.S., B.D., S.J.S., and N.S.; Methodology, D.M.W., S.P., J.C., J.-Y.A., H.A.-B., J.-L.T., K.A.A., D.R.O., I.A.G., K.R., M.W.S., B.D., S.J.S., and N.S.; Software, D.M.W., J.-Y.A., C.D., L.L., M.C.G., and S.J.S.; Validation, D.M.W., S.P., J.C., J.-Y.A., C.D., S.D., B.D., S.J.S., and N.S.; Formal Analysis, D.M.W., S.P., J.C., J.-Y.A., B.S., M.P., G.S., M.S.B., L.K., A.E.C., B.D., and S.J.S.; Investigation, D.M.W., S.P., J.C., J.-Y.A., B.S., B.D., S.J.S., and N.S.; Resources, S.P., J.C., Z.L., C.D., A.M.M.S, A.T.N.T., N.K., F.O.G., L.L., M.C.G., X.Z., H.A.-B., J.-L.T., K.A.A., D.R.O., I.A.G., M.E.T., S.J.S., and N.S.; Data Curation, D.M.W., S.P., J.C., C.D., L.L., M.C.G., X.Z., S.D., and S.J.S.; Statistical analysis, D.M.W., J.C., J.-Y.A., B.S., M.P., M.S.B., B.D., and S.J.S.; Writing – Original Draft, D.M.W., S.P., J.C., J.-Y.A., B.S., B.D., S.J.S., and N.S.; Writing – Review & Editing, D.M.W., S.P., J.C., J.-Y.A., B.S., M.P., Z.L., C.D., G.S., A.M.M.S., A.T.N.T., N.K., F.O.G., M.S.B., L.L., M.C.G., X.Z., S.D., L.K., A.E.C., J.D.B., H.A.-B., J.-L.T., K.A.A., D.R.O., I.A.G., N.A.Z., M.E.T., K.R., M.W.S., B.D., S.J.S., and N.S.; Visualization, D.M.W., S.P., J.C., J.-Y.A., B.S., S.J.S., and N.S.; Supervision, J.D.B., N.A.Z., M.E.T., K.R., M.W.S., B.D., S.J.S., and N.S.; Project Administration, D.M.W., S.P., S.J.S., and N.S.; Funding Acquisition, K.R., M.W.S., B.D., S.J.S., and N.S.

DECLARATION OF INTERESTS

The authors declare no competing interests.

Received: March 21, 2019
Revised: November 6, 2019
Accepted: March 16, 2020
Published: April 7, 2020

REFERENCES

Aguet, F., Brown, A.A., Castel, S.E., Davis, J.R., He, Y., Jo, B., Mohammadi, P., Park, Y., Parsana, P., Segrè, A.V., et al.; GTEx Consortium (2017). Genetic effects on gene expression across human tissues. Nature *550*, 204–213. https://doi.org/10.1038/nature24277.

Akbarian, S., Liu, C., Knowles, J.A., Vaccarino, F.M., Farnham, P.J., Crawford, G.E., Jaffe, A.E., Pinto, D., Dracheva, S., Geschwind, D.H., et al.; PsychENCODE Consortium (2015). The PsychENCODE project. Nat. Neurosci. *18*, 1707–1712.

An, J.Y., Lin, K., Zhu, L., Werling, D.M., Dong, S., Brand, H., Wang, H.Z., Zhao, X., Schwartz, G.B., Collins, R.L., et al. (2018). Genome-wide de novo risk score implicates promoter variation in autism spectrum disorder. Science *362*, eaat6576.

Anders, S., Pyl, P.T., and Huber, W. (2015). HTSeq–a Python framework to work with high-throughput sequencing data. Bioinformatics *31*, 166–169.

Bae, B.I., and Walsh, C.A. (2013). Neuroscience. What are mini-brains? Science *342*, 200–201.

Beecham, A.H., Patsopoulos, N.A., Xifara, D.K., Davis, M.F., Kemppinen, A., Cotsapas, C., Shah, T.S., Spencer, C., Booth, D., Goris, A., et al.; International Multiple Sclerosis Genetics Consortium (IMSGC); Wellcome Trust Case Control Consortium 2 (WTCCC2); International IBD Genetics Consortium (IIBDGC) (2013). Analysis of immune-related loci identifies 48 new susceptibility variants for multiple sclerosis. Nat. Genet. *45*, 1353–1360.

Birnbaum, R., and Weinberger, D.R. (2017). Genetic insights into the neurodevelopmental origins of schizophrenia. Nat. Rev. Neurosci. *18*, 727–740.

Bonneau, A., and Parmar, N. (2012). Effects of RhebL1 silencing on the mTOR pathway. Mol. Biol. Rep. *39*, 2129–2137.

BrainSeq: A Human Brain Genomics Consortium (2015). BrainSeq: Neurogenomics to Drive Novel Target Discovery for Neuropsychiatric Disorders. Neuron *88* (*6*), 1078–1083. https://doi.org/10.1016/j.neuron.2015.10.047.

Breen, G., Li, Q., Roth, B.L., O'Donnell, P., Didriksen, M., Dolmetsch, R., O'Reilly, P.F., Gaspar, H.A., Manji, H., Huebel, C., et al. (2016). Translating genome-wide association findings into new therapeutics for psychiatry. Nat. Neurosci. *19*, 1392–1396.

Chang, C.C., Chow, C.C., Tellier, L.C., Vattikuti, S., Purcell, S.M., and Lee, J.J. (2015a). Second-generation PLINK: rising to the challenge of larger and richer datasets. Gigascience *4*, 7.

Chang, J., Gilman, S.R., Chiang, A.H., Sanders, S.J., and Vitkup, D. (2015b). Genotype to phenotype relationships in autism spectrum disorders. Nat. Neurosci. *18*, 191–198.

Chang, D., Nalls, M.A., Hallgrímsdóttir, I.B., Hunkapiller, J., van der Brug, M., Cai, F., Kerchner, G.A., Ayalon, G., Bingol, B., Sheng, M., et al.; International Parkinson's Disease Genomics Consortium; 23andMe Research Team (2017). A meta-analysis of genome-wide association studies identifies 17 new Parkinson's disease risk loci. Nat. Genet. *49*, 1511–1516.

Chou, C.H., Shrestha, S., Yang, C.D., Chang, N.W., Lin, Y.L., Liao, K.W., Huang, W.C., Sun, T.H., Tu, S.J., Lee, W.H., et al. (2018). miRTarBase update 2018: a resource for experimentally validated microRNA-target interactions. Nucleic Acids Res. *46* (*D1*), D296–D302.

Colantuoni, C., Lipska, B.K., Ye, T., Hyde, T.M., Tao, R., Leek, J.T., Colantuoni, E.A., Elkahloun, A.G., Herman, M.M., Weinberger, D.R., and Kleinman, J.E. (2011). Temporal dynamics and genetic control of transcription in the human prefrontal cortex. Nature *478*, 519–523.

Cornish, A.J., and Markowetz, F. (2014). SANTA: quantifying the functional content of molecular networks. PLoS Comput. Biol. *10*, e1003808.

Courchesne, E., Pierce, K., Schumann, C.M., Redcay, E., Buckwalter, J.A., Kennedy, D.P., and Morgan, J. (2007). Mapping early brain development in autism. Neuron *56*, 399–413.

de Leeuw, C.A., Mooij, J.M., Heskes, T., and Posthuma, D. (2015). MAGMA: generalized gene-set analysis of GWAS data. PLoS Comput. Biol. *11*, e1004219.

Deciphering Developmental Disorders Study (2017). Prevalence and architecture of de novo mutations in developmental disorders. Nature *542*, 433–438.

Demontis, D., Walters, R.K., Martin, J., Mattheisen, M., Als, T.D., Agerbo, E., Baldursson, G., Belliveau, R., Bybjerg-Grauholm, J., Bækvad-Hansen, M., et al.; ADHD Working Group of the Psychiatric Genomics Consortium (PGC); Early Lifecourse & Genetic Epidemiology (EAGLE) Consortium; 23andMe Research Team (2019). Discovery of the first genome-wide significant risk loci for attention deficit/hyperactivity disorder. Nat. Genet. *51*, 63–75.

Devlin, B., and Roeder, K. (1999). Genomic control for association studies. Biometrics *55*, 997–1004.

Dobbyn, A., Huckins, L.M., Boocock, J., Sloofman, L.G., Glicksberg, B.S., Giambartolomei, C., Hoffman, G.E., Perumal, T.M., Girdhar, K., Jiang, Y., et al.; CommonMind Consortium (2018). Landscape of Conditional eQTL in Dorsolateral Prefrontal Cortex and Co-localization with Schizophrenia GWAS. Am. J. Hum. Genet. *102*, 1169–1184.

Dobin, A., Davis, C.A., Schlesinger, F., Drenkow, J., Zaleski, C., Jha, S., Batut, P., Chaisson, M., and Gingeras, T.R. (2013). STAR: ultrafast universal RNA-seq aligner. Bioinformatics *29*, 15–21.

Domazet-Lošo, T., and Tautz, D. (2010). A phylogenetically based transcriptome age index mirrors ontogenetic divergence patterns. Nature *468*, 815–818.

Ecker, C., Bookheimer, S.Y., and Murphy, D.G. (2015). Neuroimaging in autism spectrum disorder: brain structure and function across the lifespan. Lancet Neurol. *14*, 1121–1134.

Elliott, L.T., Sharp, K., Alfaro-Almagro, F., Shi, S., Miller, K.L., Douaud, G., Marchini, J., and Smith, S.M. (2018). Genome-wide association studies of brain imaging phenotypes in UK Biobank. Nature *562*, 210–216.

Freed, D.N., Aldana, R., Weber, J.A., and Edwards, J.S. (2017). The Sentieon Genomics Tools - A fast and accurate solution to variant calling from next-generation sequence data. bioRxiv. https://doi.org/10.1101/115717.

Fromer, M., Roussos, P., Sieberts, S.K., Johnson, J.S., Kavanagh, D.H., Perumal, T.M., Ruderfer, D.M., Oh, E.C., Topol, A., Shah, H.R., et al. (2016). Gene expression elucidates functional impact of polygenic risk for schizophrenia. Nat. Neurosci. *19*, 1442–1453.

Geschwind, D.H., and Flint, J. (2015). Genetics and genomics of psychiatric disease. Science *349*, 1489–1494.

Giambartolomei, C., Vukcevic, D., Schadt, E.E., Franke, L., Hingorani, A.D., Wallace, C., and Plagnol, V. (2014). Bayesian test for colocalisation between pairs of genetic association studies using summary statistics. PLoS Genet. *10*, e1004383.

Gibbs, J.R., van der Brug, M.P., Hernandez, D.G., Traynor, B.J., Nalls, M.A., Lai, S.L., Arepalli, S., Dillman, A., Rafferty, I.P., Troncoso, J., et al. (2010). Abundant quantitative trait loci exist for DNA methylation and gene expression in human brain. PLoS Genet. *6*, e1000952.

Gogarten, S.M., Bhangale, T., Conomos, M.P., Laurie, C.A., McHugh, C.P., Painter, I., Zheng, X., Crosslin, D.R., Levine, D., Lumley, T., et al. (2012). GWASTools: an R/Bioconductor package for quality control and analysis of genome-wide association studies. Bioinformatics *28*, 3329–3331.

Grove, J., Ripke, S., Als, T.D., Mattheisen, M., Walters, R., Won, H., Pallesen, J., Agerbo, E., Andreassen, O.A., Anney, R., et al. (2019). Identification of common genetic risk variants for autism spectrum disorder. Nat. Genet. *51*, 431–444.

GTEx Consortium (2015). Human genomics. The Genotype-Tissue Expression (GTEx) pilot analysis: multitissue gene regulation in humans. Science *348*, 648–660.

Gulsuner, S., Walsh, T., Watts, A.C., Lee, M.K., Thornton, A.M., Casadei, S., Rippey, C., Shahin, H., Nimgaonkar, V.L., Go, R.C., et al.; Consortium on the Genetics of Schizophrenia (COGS); PAARTNERS Study Group (2013). Spatial and temporal mapping of de novo mutations in schizophrenia to a fetal prefrontal cortical network. Cell *154*, 518–529.

Harrow, J., Frankish, A., Gonzalez, J.M., Tapanari, E., Diekhans, M., Kokocinski, F., Aken, B.L., Barrell, D., Zadissa, A., Searle, S., et al. (2012). GENCODE: the reference human genome annotation for The ENCODE Project. Genome Res. *22*, 1760–1774.

Heinzen, E.L., Ge, D., Cronin, K.D., Maia, J.M., Shianna, K.V., Gabriel, W.N., Welsh-Bohmer, K.A., Hulette, C.M., Denny, T.N., and Goldstein, D.B. (2008). Tissue-specific genetic control of splicing: implications for the study of complex traits. PLoS Biol. *6*, e1.

Heyne, H.O., Singh, T., Stamberger, H., Abou Jamra, R., Caglayan, H., Craiu, D., De Jonghe, P., Guerrini, R., Helbig, K.L., Koeleman, B.P.C., et al.; EuroEPINOMICS RES Consortium (2018). De novo variants in neurodevelopmental disorders with epilepsy. Nat. Genet. *50*, 1048–1053.

Hu, W.F., Chahrour, M.H., and Walsh, C.A. (2014). The diverse genetic landscape of neurodevelopmental disorders. Annu. Rev. Genomics Hum. Genet. *15*, 195–213.

Huber, W., Carey, V.J., Gentleman, R., Anders, S., Carlson, M., Carvalho, B.S., Bravo, H.C., Davis, S., Gatto, L., Girke, T., et al. (2015). Orchestrating high-throughput genomic analysis with Bioconductor. Mat. Methods *12*, 115–121.

Insel, T.R. (2010). Rethinking schizophrenia. Nature *468*, 187–193.

Jaffe, A.E., Straub, R.E., Shin, J.H., Tao, R., Gao, Y., Collado-Torres, L., Kam-Thong, T., Xi, H.S., Quan, J., Chen, Q., et al.; BrainSeq Consortium (2018). Developmental and genetic regulation of the human cortex transcriptome illuminate schizophrenia pathogenesis. Nat. Neurosci. *21*, 1117–1125.

Jones, B.L., Nagin, D.S., and Roeder, K. (2001). A SAS Procedure Based on Mixture Models for Estimating Developmental Trajectories. Sociol. Methods Res. *29*, 374–393.

Jun, G., Flickinger, M., Hetrick, K.N., Romm, J.M., Doheny, K.F., Abecasis, G.R., Boehnke, M., and Kang, H.M. (2012). Detecting and estimating contamination of human DNA samples in sequencing and array-based genotype data. Am. J. Hum. Genet. *91*, 839–848.

Kalinka, A.T., Varga, K.M., Gerrard, D.T., Preibisch, S., Corcoran, D.L., Jarrells, J., Ohler, U., Bergman, C.M., and Tomancak, P. (2010). Gene expression divergence recapitulates the developmental hourglass model. Nature *468*, 811–814.

Kang, H.J., Kawasawa, Y.I., Cheng, F., Zhu, Y., Xu, X., Li, M., Sousa, A.M., Pletikos, M., Meyer, K.A., Sedmak, G., et al. (2011). Spatio-temporal transcriptome of the human brain. Nature *478*, 483–489.

Kent, W.J., Sugnet, C.W., Furey, T.S., Roskin, K.M., Pringle, T.H., Zahler, A.M., and Haussler, D. (2002). The human genome browser at UCSC. Genome Res. *12*, 996–1006.

Keshavan, M.S., Giedd, J., Lau, J.Y., Lewis, D.A., and Paus, T. (2014). Changes in the adolescent brain and the pathophysiology of psychotic disorders. Lancet Psychiatry *1*, 549–558.

Kosmicki, J.A., Churchhouse, C.L., Rivas, M.A., and Neale, B.M. (2016). Discovery of rare variants for complex phenotypes. Hum. Genet. *135*, 625–634.

Kryuchkova-Mostacci, N., and Robinson-Rechavi, M. (2017). A benchmark of gene expression tissue-specificity metrics. Brief. Bioinform. *18*, 205–214.

Kundaje, A., Meuleman, W., Ernst, J., Bilenky, M., Yen, A., Heravi-Moussavi, A., Kheradpour, P., Zhang, Z., Wang, J., Ziller, M.J., et al.; Roadmap Epigenomics Consortium (2015). Integrative analysis of 111 reference human epigenomes. Nature *518*, 317–330.

Lachmann, A., Xu, H., Krishnan, J., Berger, S.I., Mazloom, A.R., and Ma'ayan, A. (2010). ChEA: transcription factor regulation inferred from integrating genome-wide ChIP-X experiments. Bioinformatics *26*, 2438–2444.

Lambert, J.C., Ibrahim-Verbaas, C.A., Harold, D., Naj, A.C., Sims, R., Bellenguez, C., DeStafano, A.L., Bis, J.C., Beecham, G.W., Grenier-Boley, B., et al.; European Alzheimer's Disease Initiative (EADI); Genetic and Environmental Risk in Alzheimer's Disease; Alzheimer's Disease Genetic Consortium; Cohorts for Heart and Aging Research in Genomic Epidemiology (2013). Meta-analysis of 74,046 individuals identifies 11 new susceptibility loci for Alzheimer's disease. Nat. Genet. *45*, 1452–1458.

Langfelder, P., and Horvath, S. (2007). Eigengene networks for studying the relationships between co-expression modules. BMC Syst. Biol. *1*, 54.

Langfelder, P., and Horvath, S. (2008). WGCNA: an R package for weighted correlation network analysis. BMC Bioinformatics *9*, 559.

Langfelder, P., Luo, R., Oldham, M.C., and Horvath, S. (2011). Is my network module preserved and reproducible? PLoS Comput. Biol. *7*, e1001057.

Law, C.W., Chen, Y., Shi, W., and Smyth, G.K. (2014). voom: Precision weights unlock linear model analysis tools for RNA-seq read counts. Genome Biol. *15*, R29.

Lee, J.J., Wedow, R., Okbay, A., Kong, E., Maghzian, O., Zacher, M., Nguyen-Viet, T.A., Bowers, P., Sidorenko, J., Karlsson Linnér, R., et al.; 23andMe Research Team; COGENT (Cognitive Genomics Consortium); Social Science Genetic Association Consortium (2018). Gene discovery and polygenic prediction from a genome-wide association study of educational attainment in 1.1 million individuals. Nat. Genet. *50*, 1112–1121.

Leek, J.T., Johnson, W.E., Parker, H.S., Jaffe, A.E., and Storey, J.D. (2012). The sva package for removing batch effects and other unwanted variation in high-throughput experiments. Bioinformatics *28*, 882–883.

Lek, M., Karczewski, K.J., Minikel, E.V., Samocha, K.E., Banks, E., Fennell, T., O'Donnell-Luria, A.H., Ware, J.S., Hill, A.J., Cummings, B.B., et al.; Exome Aggregation Consortium (2016). Analysis of protein-coding genetic variation in 60,706 humans. Nature *536*, 285–291.

Li, H. (2014). Toward better understanding of artifacts in variant calling from high-coverage samples. Bioinformatics *30*, 2843–2851.

Li, H., and Durbin, R. (2009). Fast and accurate short read alignment with Burrows-Wheeler transform. Bioinformatics *25*, 1754–1760.

Li, M., Santpere, G., Imamura Kawasawa, Y., Evgrafov, O.V., Gulden, F.O., Pochareddy, S., Sunkin, S.M., Li, Z., Shin, Y., Zhu, Y., et al.; BrainSpan Consortium; PsychENCODE Consortium; PsychENCODE Developmental Subgroup (2018). Integrative functional genomic analysis of human brain development and neuropsychiatric risks. Science *362*, eaat7615.

Lipton, J.O., and Sahin, M. (2014). The neurology of mTOR. Neuron *84*, 275–291.

Liu, C., Cheng, L., Badner, J.A., Zhang, D., Craig, D.W., Redman, M., and Gershon, E.S. (2010). Whole-genome association mapping of gene expression in the human prefrontal cortex. Mol. Psychiatry *15*, 779–784.

Love, M.I., Huber, W., and Anders, S. (2014). Moderated estimation of fold change and dispersion for RNA-seq data with DESeq2. Genome Biol. *15*, 550.

Lui, J.H., Hansen, D.V., and Kriegstein, A.R. (2011). Development and evolution of the human neocortex. Cell *146*, 18–36.

Matys, V., Fricke, E., Geffers, R., Gössling, E., Haubrock, M., Hehl, R., Hornischer, K., Karas, D., Kel, A.E., Kel-Margoulis, O.V., et al. (2003). TRANSFAC: transcriptional regulation, from patterns to profiles. Nucleic Acids Res. *31*, 374–378.

McCarroll, S.A., and Hyman, S.E. (2013). Progress in the genetics of polygenic brain disorders: significant new challenges for neurobiology. Neuron *80*, 578–587.

McKenna, A., Hanna, M., Banks, E., Sivachenko, A., Cibulskis, K., Kernytsky, A., Garimella, K., Altshuler, D., Gabriel, S., Daly, M., and DePristo, M.A. (2010). The Genome Analysis Toolkit: a MapReduce framework for analyzing next-generation DNA sequencing data. Genome Res. *20*, 1297–1303.

McLaren, W., Gil, L., Hunt, S.E., Riat, H.S., Ritchie, G.R., Thormann, A., Flicek, P., and Cunningham, F. (2016). The Ensembl Variant Effect Predictor. Genome Biol. *17*, 122.

Mostafavi, S., Battle, A., Zhu, X., Urban, A.E., Levinson, D., Montgomery, S.B., and Koller, D. (2013). Normalizing RNA-sequencing data by modeling hidden covariates with prior knowledge. PLoS ONE *8*, e68141.

Myers, A.J., Gibbs, J.R., Webster, J.A., Rohrer, K., Zhao, A., Marlowe, L., Kaleem, M., Leung, D., Bryden, L., Nath, P., et al. (2007). A survey of genetic human cortical gene expression. Nat. Genet. *39*, 1494–1499.

Network and Pathway Analysis Subgroup of the Psychiatric Genomics Consortium (2015). Corrigendum: Psychiatric genome-wide association study analyses implicate neuronal, immune and histone pathways. Nat. Neurosci. *18*, 926.

Nicolae, D.L., Gamazon, E., Zhang, W., Duan, S., Dolan, M.E., and Cox, N.J. (2010). Trait-associated SNPs are more likely to be eQTLs: annotation to enhance discovery from GWAS. PLoS Genet. *6*, e1000888.

Nowakowski, T.J., Bhaduri, A., Pollen, A.A., Alvarado, B., Mostajo-Radji, M.A., Di Lullo, E., Haeussler, M., Sandoval-Espinosa, C., Liu, S.J., Velmeshev, D., et al. (2017). Spatiotemporal gene expression trajectories reveal developmental hierarchies of the human cortex. Science *358*, 1318–1323.

O'Brien, H.E., Hannon, E., Hill, M.J., Toste, C.C., Robertson, M.J., Morgan, J.E., McLaughlin, G., Lewis, C.M., Schalkwyk, L.C., Hall, L.S., et al. (2018). Expression quantitative trait loci in the developing human brain and their enrichment in neuropsychiatric disorders. Genome Biol. *19*, 194.

Parikshak, N.N., Luo, R., Zhang, A., Won, H., Lowe, J.K., Chandran, V., Horvath, S., and Geschwind, D.H. (2013). Integrative functional genomic analyses implicate specific molecular pathways and circuits in autism. Cell *155*, 1008–1021.

Pedersen, B.S., and Quinlan, A.R. (2017). Who's Who? Detecting and Resolving Sample Anomalies in Human DNA Sequencing Studies with Peddy. Am. J. Hum. Genet. *100*, 406–413.

Pletikos, M., Sousa, A.M., Sedmak, G., Meyer, K.A., Zhu, Y., Cheng, F., Li, M., Kawasawa, Y.I., and Sestan, N. (2014). Temporal specification and bilaterality of human neocortical topographic gene expression. Neuron *81*, 321–332.

Pruim, R.J., Welch, R.P., Sanna, S., Teslovich, T.M., Chines, P.S., Gliedt, T.P., Boehnke, M., Abecasis, G.R., and Willer, C.J. (2010). LocusZoom: regional visualization of genome-wide association scan results. Bioinformatics *26*, 2336–2337.

Quinlan, A.R., and Hall, I.M. (2010). BEDTools: a flexible suite of utilities for comparing genomic features. Bioinformatics *26*, 841–842.

Regier, A.A., Farjoun, Y., Larson, D.E., Krasheninina, O., Kang, H.M., Howrigan, D.P., Chen, B.J., Kher, M., Banks, E., Ames, D.C., et al. (2018). Functional equivalence of genome sequencing analysis pipelines enables harmonized variant calling across human genetics projects. Nat. Commun. *9*, 4038.

Reilly, S.K., Yin, J., Ayoub, A.E., Emera, D., Leng, J., Cotney, J., Sarro, R., Rakic, P., and Noonan, J.P. (2015). Evolutionary genomics. Evolutionary changes in promoter and enhancer activity during human corticogenesis. Science *347*, 1155–1159.

Reimand, J., Arak, T., and Vilo, J. (2011). g:Profiler–a web server for functional interpretation of gene lists (2011 update). Nucleic Acids Res. *39*, W307-15.

Ritchie, M.E., Phipson, B., Wu, D., Hu, Y., Law, C.W., Shi, W., and Smyth, G.K. (2015). limma powers differential expression analyses for RNA-sequencing and microarray studies. Nucleic Acids Res. *43*, e47.

Roeder, K., Lynch, K.G., and Nagin, D.S. (1999). Modeling Uncertainty in Latent Class Membership: A Case Study in Criminology. J. Am. Stat. Assoc. *94*, 766–776.

Rosti, R.O., Sadek, A.A., Vaux, K.K., and Gleeson, J.G. (2014). The genetic landscape of autism spectrum disorders. Dev. Med. Child Neurol. *56*, 12–18.

Sanders, S.J., He, X., Willsey, A.J., Ercan-Sencicek, A.G., Samocha, K.E., Cicek, A.E., Murtha, M.T., Bal, V.H., Bishop, S.L., Dong, S., et al.; Autism Sequencing Consortium (2015). Insights into Autism Spectrum Disorder Genomic Architecture and Biology from 71 Risk Loci. Neuron *87*, 1215–1233.

Sanders, S.J., Neale, B.M., Huang, H., Werling, D.M., An, J.Y., Dong, S., Abecasis, G., Arguello, P.A., Blangero, J., Boehnke, M., et al.; Whole Genome Sequencing for Psychiatric Disorders (WGSPD) (2017). Whole genome sequencing in psychiatric disorders: the WGSPD consortium. Nat. Neurosci. *20*, 1661–1668.

Satterstrom, F.K., Kosmicki, J.A., Wang, J., Breen, M.S., De Rubeis, S., An, J.Y., Peng, M., Collins, R., Grove, J., Klei, L., et al. (2020). Large-Scale Exome Sequencing Study Implicates Both Developmental and Functional Changes in the Neurobiology of Autism. Cell *180*, 568–584.e23.

Schizophrenia Working Group of the Psychiatric Genomics Consortium (2014). Biological insights from 108 schizophrenia-associated genetic loci. Nature *511*, 421–427.

Schmidt, E.M., Zhang, J., Zhou, W., Chen, J., Mohlke, K.L., Chen, Y.E., and Willer, C.J. (2015). GREGOR: evaluating global enrichment of trait-associated variants in epigenomic features using a systematic, data-driven approach. Bioinformatics *31*, 2601–2606.

Sestan, N., and State, M.W. (2018). Lost in Translation: Traversing the Complex Path from Genomics to Therapeutics in Autism Spectrum Disorder. Neuron *100*, 406–423.

Shaw, P., Gogtay, N., and Rapoport, J. (2010). Childhood psychiatric disorders as anomalies in neurodevelopmental trajectories. Hum. Brain Mapp. *31*, 917–925.

Silbereis, J.C., Pochareddy, S., Zhu, Y., Li, M., and Sestan, N. (2016). The Cellular and Molecular Landscapes of the Developing Human Central Nervous System. Neuron *89*, 248–268.

Stark, C., Breitkreutz, B.J., Reguly, T., Boucher, L., Breitkreutz, A., and Tyers, M. (2006). BioGRID: a general repository for interaction datasets. Nucleic Acids Res. *34*, D535–D539.

Tang, S.X., and Gur, R.E. (2018). Longitudinal perspectives on the psychosis spectrum in 22q11.2 deletion syndrome. Am. J. Med. Genet. A. *176*, 2192–2202.

Tau, G.Z., and Peterson, B.S. (2010). Normal development of brain circuits. Neuropsychopharmacology *35*, 147–168.

Turner, T.N., and Eichler, E.E. (2019). The Role of De Novo Noncoding Regulatory Mutations in Neurodevelopmental Disorders. Trends Neurosci. *42*, 115–127.

Velmeshev, D., Schirmer, L., Jung, D., Haeussler, M., Perez, Y., Mayer, S., Bhaduri, A., Goyal, N., Rowitch, D.H., and Kriegstein, A.R. (2019). Single-cell genomics identifies cell type-specific molecular changes in autism. Science *364*, 685–689.

Walker, R.L., Ramaswami, G., Hartl, C., Mancuso, N., Gandal, M.J., de la Torre-Ubieta, L., Pasaniuc, B., Stein, J.L., and Geschwind, D.H. (2019). Genetic Control of Expression and Splicing in Developing Human Brain Informs Disease Mechanisms. Cell *179*, 750–771.e722.

Wang, D., Liu, S., Warrell, J., Won, H., Shi, X., Navarro, F.C.P., Clarke, D., Gu, M., Emani, P., Yang, Y.T., et al.; PsychENCODE Consortium (2018). Comprehensive functional genomic resource and integrative model for the human brain. Science *362*, 6420.

Werling, D.M., Brand, H., An, J.Y., Stone, M.R., Zhu, L., Glessner, J.T., Collins, R.L., Dong, S., Layer, R.M., Markenscoff-Papadimitriou, E., et al. (2018). An

analytical framework for whole-genome sequence association studies and its implications for autism spectrum disorder. Nat. Genet. *50*, 727–736.

Willer, C.J., Schmidt, E.M., Sengupta, S., Peloso, G.M., Gustafsson, S., Kanoni, S., Ganna, A., Chen, J., Buchkovich, M.L., Mora, S., et al.; Global Lipids Genetics Consortium (2013). Discovery and refinement of loci associated with lipid levels. Nat. Genet. *45*, 1274–1283.

Willsey, A.J., Sanders, S.J., Li, M., Dong, S., Tebbenkamp, A.T., Muhle, R.A., Reilly, S.K., Lin, L., Fertuzinhos, S., Miller, J.A., et al. (2013). Coexpression networks implicate human midfetal deep cortical projection neurons in the pathogenesis of autism. Cell *155*, 997–1007.

Winter, A.G., Wildenhain, J., and Tyers, M. (2011). BioGRID REST Service, BiogridPlugin2 and BioGRID WebGraph: new tools for access to interaction data at BioGRID. Bioinformatics *27*, 1043–1044.

Wood, A.R., Esko, T., Yang, J., Vedantam, S., Pers, T.H., Gustafsson, S., Chu, A.Y., Estrada, K., Luan, J., Kutalik, Z., et al.; Electronic Medical Records and Genomics (eMEMERGEGE) Consortium; MIGen Consortium; PAGEGE Consortium; LifeLines Cohort Study (2014). Defining the role of common variation in the genomic and biological architecture of adult human height. Nat. Genet. *46*, 1173–1186.

Wray, N.R., Ripke, S., Mattheisen, M., Trzaskowski, M., Byrne, E.M., Abdellaoui, A., Adams, M.J., Agerbo, E., Air, T.M., Andlauer, T.M.F., et al.; eQTLGen; 23andMe; Major Depressive Disorder Working Group of the Psychiatric Genomics Consortium (2018). Genome-wide association analyses identify 44 risk variants and refine the genetic architecture of major depression. Nat. Genet. *50*, 668–681.

Xu, X., Wells, A.B., O'Brien, D.R., Nehorai, A., and Dougherty, J.D. (2014). Cell type-specific expression analysis to identify putative cellular mechanisms for neurogenetic disorders. J. Neurosci. *34*, 1420–1431.

Zhu, Y., Sousa, A.M.M., Gao, T., Skarica, M., Li, M., Santpere, G., Esteller-Cucala, P., Juan, D., Ferrández-Peral, L., Gulden, F.O., et al. (2018). Spatiotemporal transcriptomic divergence across human and macaque brain development. Science *362*, 362.

STAR★METHODS

KEY RESOURCES TABLE

REAGENT or RESOURCE	SOURCE	IDENTIFIER
Biological Samples		
See Table S1 for a list of human post-mortem tissue included in the study.		N/A
Chemicals, Peptides, and Recombinant Proteins		
Agencourt AMPure XP beads	Beckman Coulter	A63881
RNAClean XP beads	Beckman Coulter	A63987
RNA ScreenTape	Agilent	5067-5576
RNA ScreenTape Sample Buffer	Agilent	5067-5577
D1000 ScreenTape	Agilent	5067-5582
D1000 Sample Buffer	Agilent	5067-5602
Stainless Steel Beads 0.9 – 2.0 mm blend	Next Advance	SSB14B
Critical Commercial Assays		
mirVana miRNA Isolation Kit, with phenol	Thermo Fisher Scientific	AM1560
TURBO DNA-free Kit	Thermo Fisher Scientific	AM1907
TruSeq Stranded Total RNA HT Sample Prep Kit with Ribo-Zero Gold kit	Illumina	20020599
QIAamp DNA Mini Kit (250)	QIAGEN	51306
Deposited Data		
Raw WGS data	https://www.synapse.org/#!Synapse:syn4921369	syn21557948
Processed WGS data	https://www.synapse.org/#!Synapse:syn4921369	syn21557948
Raw RNA-seq data	https://www.synapse.org/#!Synapse:syn4921369	syn21557948
Processed RNA-seq data	https://www.synapse.org/#!Synapse:syn4921369	syn21557948
Software and Algorithms		
HTSeq v.0.6.0.	Anders et al., 2015	https://htseq.readthedocs.io/en/master/
STAR v.2.4.2a	Dobin et al., 2013	https://github.com/alexdobin/STAR
limma v.3.36.5	Ritchie et al., 2015	http://bioconductor.org/packages/release/bioc/html/limma.html
HCP	Mostafavi et al., 2013	https://github.com/mvaniterson/Rhcpp
SVA v. 3.28.0	Leek et al., 2012	https://www.bioconductor.org/packages/release/bioc/html/sva.html
rsq v.1.1	Zhang Lab, Purdue	https://cran.r-project.org/web/packages/rsq/index.html
igraph v.1.2.2	RStudio, Inc., Boston	https://cran.r-project.org/web/packages/igraph/index.html
SANTA v.2.14.0	Cornish and Markowetz, 2014	https://bioconductor.org/packages/release/bioc/html/SANTA.html
WGCNA v 1.63	Langfelder and Horvath, 2008	https://horvath.genetics.ucla.edu/html/CoexpressionNetwork/Rpackages/WGCNA/
BWA v0.7.15	Li and Durbin, 2009	https://github.com/lh3/bwa/releases
Picard v2.17.5 for sorting & removing duplicate reads, v2.18.1 for checking sample sequencing depth	Broad Institute, Boston	https://github.com/broadinstitute/picard/

(*Continued on next page*)

Continued

REAGENT or RESOURCE	SOURCE	IDENTIFIER
GenomeAnalysisToolKit GATK v3.8-0-ge9d806836	McKenna et al., 2010	https://github.com/broadgsa/gatk
Sentieon v201711.01	Freed et al., 2017	https://www.sentieon.com/products/
VerifyBamId v1.1.3	Jun et al., 2012	https://github.com/statgen/verifyBamID/
Peddy v0.3.2	Pedersen and Quinlan, 2017	https://github.com/brentp/peddy
Hail v0.1	Github	https://github.com/hail-is/hail
coloc	Giambartolomei et al., 2014	http://cran.r-project.org/web/packages/coloc
MAGMA v1.07b	de Leeuw et al., 2015	https://ctg.cncr.nl/software/magma
gProfileR v0.6.7	Reimand et al., 2011	https://github.com/cran/gProfileR/releases
GREGOR	Schmidt et al., 2015	http://csg.sph.umich.edu/GREGOR/index.php/site/download
Whole-genome analysis pipeline	Sanders Lab, UCSF	https://github.com/sanderslab/psychcore-compute-platform
Other		
GENCODE annotation v21 (GRCh38)	Harrow et al., 2012	https://www.gencodegenes.org/human/release_21.html
HUGO Gene Nomenclature Committee Complete dataset (2018)	European Bioinformatics Institute, Cambridge, UK	ftp://ftp.ebi.ac.uk/pub/databases/genenames/new/tsv/hgnc_complete_set.txt
BioGRID (v3.4.132)	Stark et al., 2006	https://downloads.thebiogrid.org/BioGRID/Release-Archive/BIOGRID-3.4.132/
ENSEMBLE VEP (v90)	McLaren et al., 2016	https://github.com/Ensembl/ensembl-vep/releases

LEAD CONTACT AND MATERIALS AVAILABILITY

Further information and requests for resources and reagents should be directed to and will be fulfilled by Stephan Sanders (stephan.sanders@ucsf.edu).

This study did not generate new unique reagents.

EXPERIMENTAL MODEL AND SUBJECT DETAILS

This study was conducted using postmortem human brain specimens from tissue collections at the Department of Neuroscience at Yale University School of Medicine. Additional specimens were procured from the Birth Defects Research Laboratory at the University of Washington, Advanced Bioscience Resources Inc., Human Brain Collection Core (HBCC), the Brain and Tissue Bank at the University of Maryland, the MRC-Wellcome Trust Human Developmental Biology Resource at the Institute of Human Genetics, University of Newcastle, UK, and the Human Fetal Tissue Repository at the Albert Einstein College of Medicine (AECOM). Tissue was collected after obtaining parental or next of kin consent and with approval by the institutional review boards at the Yale University School of Medicine, the National Institutes of Health, and at each institution from which tissue specimens were obtained. Tissue was handled in accordance with ethical guidelines and regulations for the research use of human brain tissue set forth by the NIH (https://oir.nih.gov/sites/default/files/uploads/sourcebook/documents/ethical_conduct/guidelines-biospecimen.pdf and the WMA Declaration of Helsinki (https://www.wma.net/policies-post/wma-declaration-of-helsinki-ethical-principles-for-medical-research-involving-human-subjects/).

All available non-identifying information was recorded for each specimen in Table S1. In total, 176 postmortem brain specimens (104 male, 72 female; postmortem interval of 21.7 ± 15.9 (mean ± SD) hours and pH, 6.41 ± 0.35) ranging in age from 6 post-conception weeks to 20 postnatal years (Figure 1; Table S1) were included in this study. Fetal age was extrapolated based on the date of the mother's last menstruation, characteristics of the fetus noted upon ultrasonography scanning, foot length of the fetus, and visual inspection. The postmortem interval (PMI) was defined as hours between time of death and time when tissue samples were frozen.

METHOD DETAILS

Tissue dissection

Tissue was dissected as described previously (Kang et al., 2011). Samples collected from 6 – 9 post-conception weeks specimens contained the entire thickness of the cerebral wall. Samples collected from 12 to 22 post-conception weeks specimens contained the

cortical plate. Samples from 35 post-conception weeks to 20 postnatal years were dissected such that the entire gray matter (layer 1-6) and part of the underlying subplate (4 – 12 postnatal months) or white matter (1 – 20 postnatal years) were collected.

RNA extraction and quality assessment

Total RNA was extracted using mirVana kit (Ambion) with some modificationsto the manufacturer's protocol, as described below. Each tissue sample was pulverized with liquid nitrogen in a prechilled mortar and pestle and transferred to a chilled safe-lock microcentrifuge tube (Eppendorf). Per tissue mass, equal mass of chilled stainless-steel beads (Next Advance, catalog # SSB14B) along with one volume of lysis/binding buffer were added. Tissue was homogenized for 1 min in Bullet Blender (Next Advance) and incubated at 37°C for 1 min. Another nine volumes of the lysis/binding buffer were added, homogenized for 1 min, and incubated at 37°C for 2 min. One-tenth volume of miRNA Homogenate Additive was added, and extraction was carried out according to the manufacturer's protocol. RNA was treated with DNase using TURBO DNA-free Kit (Ambion/Life Technologies) and RNA integrity was measured using Agilent 2200 TapeStation System.

RNA-seq library preparation and sequencing

Barcoded libraries for RNA-seq were prepared with 5ng of RNA using TruSeq Stranded Total RNA HT Sample Prep Kit with Ribo-Zero Gold kit (Illumina) per manufacturer's protocol. Paired-end sequencing (100 bp x 2) was performed on HiSeq 4000 sequencers (Illumina) at Yale Center for Genome Analysis.

DNA extraction

Genomic DNA was isolated using the QIAamp DNA Mini Kit (QIAGEN). In detail, approximately 25 mg of brain tissue was transferred to a chilled safe-lock microcentrifuge tube (Eppendorf) and equal mass of chilled stainless-steel beads (Next Advance, catalog # SSB14B) along with 90 μl of buffer ATL were added. Tissue was homogenized for 1 min in Bullet Blender (Next Advance) and incubated at 37°C for 1 min. Another 90 ul of buffer ATL was added and blended for an additional minute. After incubation on ice for 5 min, tubes were gently centrifuged to collect beads at the bottom. Supernatant was transferred to a new tube and 20 μl of Proteinase K was added. Sample was incubated at 56°C for 3 hours in a shaking heat block. After incubation, genomic DNA was further purified following the manufacturer's protocol. DNA was eluted in nuclease free water and concentration was estimated by nanodrop.

Whole-genome sequencing

DNA library preparation and sequencing were carried out at GENEWIZ (New Jersey). Before library preparation, the concentration of the DNA was measured using a fluorescent assay and DNA quality was assessed by visualization on agarose gels. PCR-free DNA library preparation was performed and resulting libraries were sequenced at 2x150 bp to achieve mean coverage of 30x (Table S1).

QUANTIFICATION AND STATISTICAL ANALYSIS

WGS variant calling

Using the pipeline from the Centers for the Common Disease Genomics project (Regier et al., 2018), FASTQ reads were aligned to the GRCh38 reference from the 1000 Genomes Project using BWA-MEM version 0.7.15 (Li and Durbin, 2009). Reads were sorted and duplicates were removed with Picard, version 2.17.5 (https://github.com/broadinstitute/picard/); base quality score recalibration was then performed with the Genome Analysis Toolkit (GATK), v3.8-0-ge9d806836 (McKenna et al., 2010). Variant calling and joint genotyping were done with the Haplotyper and Genotyper tools from Sentieon v201711.01, a toolkit containing modules that are mathematically equivalent to their counterparts in the GATK (Freed et al., 2017). SNP and indel recalibration were performed on the joint genotyped VCF file. Variant Quality Score Recalibration (VQSR) metrics were created from a training set of highly validated variant resources: dbSNP build 138, HapMap 3.3, 1000 Genomes OMNI 2.5, and 1000 Genomes Phase 1. For the following analyses, we excluded: variant calls with any VQSR tranches (keeping "PASS" only), variants located in low-complexity regions (Li, 2014), variants located on non-canonical chromosomes (decoy chromosomes or contigs), indels > 50 bp, single nucleotide variants (SNVs) with allele balance > 0.78 or < 0.22 (indels > 0.8 or < 0.2), variants with < 90% call rate, and variants and genotypes that did not meet high quality thresholds as identified in an ROC-based optimization procedure using family-based WGS data (Werling et al., 2018).

Variants with a minor allele frequency of ≥ 5% in both the prenatal (periods 1-6; N = 112) and postnatal (periods 8-12; N = 60) samples and Hardy Weinberg equilibrium p value ≥ $1x10^{-12}$, were included in downstream expression quantitative trait locus (eQTL) analysis (N = 6,573,196 variants). For annotation and subsequent analyses, we converted the final VCF into Variant Dataset Format using Hail version 0.1. SNPs and insertions/deletions (up to 50bp) annotation based on the GENCODE comprehensive version 21 (Harrow et al., 2012) using Ensemble VEP version 90 (McLaren et al., 2016).

RNA-seq alignment and gene-level read count quantification

RNA-seq reads were aligned to the human genome (hg38/GRCh38) using STAR aligner (Dobin et al., 2013) and gene-level read counts were calculated using HTSeq (Anders et al., 2015) based on GENCODE v21 annotation (Harrow et al., 2012).

Read counts per gene were then converted to counts per million (CPM), which were logarithmically scaled to base 2 (log_2CPM). Of the 60,155 genes assessed, 23,782 were defined as being cortically expressed, based on CPM ≥ 1 in at least 50% of samples of

either sex in at least one of the 12 developmental periods (Table S2). For these 23,782 genes, the median $\log_2$CPM ranged from -5.9 to 12.3, with a median of 2.3.

RNA-seq normalization and technical artifact correction

The read count data matrix of 176 samples by 60,155 genes (Table S2) was normalized as follows:

- Step 1: Read count data matrix converted to counts per million (CPM).
- Step 2: Genes with CPM $\geq$ 1 CPM in at least 50% of the samples in any one sex in any one period were included; 23,782 genes passed these criteria.
- Step 3: CPM values were transformed to $\log_2$(CPM) using the voom function in the limma R package (Law et al., 2014; Ritchie et al., 2015).
- Step 4A: In order to correct for the technical artifacts, we performed hidden covariate analysis on the residuals of the expression matrix after developmental period and sex were subtracted from the $\log_2$(CPM) data matrix using the hidden covariate analysis method (HCP) (Mostafavi et al., 2013).
- Step 4B: In parallel, we performed surrogate variable analysis (SVA) on residuals of the expression matrix after developmental period and sex were subtracted from the $\log_2$(CPM) data matrix using the SVA R package (Leek et al., 2012).
- Step 5: In each of Step 4A and 4B, we subtracted contributions from 20 hidden covariates (HCP) and 2 surrogate variables (SVA) from the $\log_2$(CPM) data matrix.

Unadjusted $\log_2$CPM gene expression data for 176 samples by 23,782 genes was used for expression trajectory (Figure 2) and WGCNA analyses (Figure 3), while the adjusted (HCP/SVA) values were used for eQTL analysis.

DATA QUALITY AND SAMPLE IDENTITY ASSESSMENT

To confirm that the WGS and RNA-seq data from each sample were of sufficient quality for downstream analysis and corresponded to the same individual, a series of quality metrics and checks were performed (Table S1).

For the WGS data, coverage metrics were assessed using PicardTools (v2.18.1). Mean coverage per sample ranged from 22.7-65.4x, with a cohort median of 31.5x. Across all samples, 92.1%–93.6% of the mapped genome was covered at 10x or greater (mean of 92.8%). The FREEMIX metric from VerifyBamId (v1.1.3; Jun et al., 2012) was used to identify samples with potential contamination, with a maximum observed FREEMIX score of 0.064, suggesting no contamination (Lek et al., 2016).

Sample identity was verified by comparing sex and genotype between the WGS and RNA-seq data. In the WGS data, sex was determined from chromosome X heterozygosity using Peddy (v0.3.2; (Pedersen and Quinlan, 2017)), with the Peddy hg19.sites converted to GRCh38 using the UCSC Genome Browser LiftOver utility. High-quality variants with an allele frequency $\geq$ 1% were exported from the VCF using Hail for input into Peddy. In the RNA-seq data, sex was determined from the expression levels of *XIST* and the 18 most highly expressed genes on chromosome Y: *KDM5D*, *DDX3Y*, *ZFY*, *TBL1Y*, *PCDH11Y*, *PRKY*, *USP9Y*, *RPS4Y1*, *TXLNGY*, *NLGN4Y*, *TTTY14*, *UTY*, *EIF1AY*, *GYG2P1*, *TTTY10*, *TTTY15*, *KALP*. Based on gene-specific expression thresholds determined by visual inspection of bi-modal expression histograms, each sample's sex was predicted according to the expression level of all 19 genes. Sex was consistent in the WGS and RNA-seq data for all 176 samples and matched the recorded sex in 132 out of 134 samples with such data (55/56 females, 77/78 males).

To confirm identity by genotype, we compared the genotypes from 289 common, coding SNPs with high fixation index (F_{ST}) (Sanders et al., 2015), called from both the WGS and RNA-seq data. Genotypes were callable in both data types for 118-206 SNPs per sample (40.8%–71.3% of 289 SNPs; median = 177, 61.2%). SNP variant genotypes were highly concordant between the WGS and RNA-seq data (median 87.4% concordance between WGS and RNA-seq for the corresponding sample; lowest concordance 73%), with corresponding samples showing higher concordance than comparisons between all discordant samples. There was no evidence of duplicate or closely related samples (SNP-based relatedness coefficients from Peddy: -0.000332 to 0.1481).

To confirm the approximate accuracy of samples' reported age, the expression level of the doublecortin gene (*DCX*) was examined. DCX is involved in neuron migration, and is expressed most strongly during prenatal development, with distinctly decreased postnatal expression. All 176 samples showed the expected DCX expression levels given samples' reported age. Similar results across all expressed genes were obtained using principal component analysis (below).

Ancestry estimation

Ancestry was estimated using principal component analysis of common SNPs and indels in the WGS data, run alongside 3,804 additional individuals of known ancestry with WGS data (parents from the Simons Simplex Collection; An et al., 2018). From 10,688,106 SNPs and indels with allele frequency $\geq$ 5% in either this dataset, one of the three batches of Simons Simplex Collection data, or GnomAD genomes, variants were pruned for independence with linkage disequilibrium $r^2 < 0.1$ and then randomly downsampled to 118,849 variants. Principal component analysis was run using Hail 0.1. The first two principal components were used to classify samples by ancestry, and the first five principal components were used as covariates in the identification of eQTL loci (Figure S1).

Using the first two principal components of SNP-based ancestry estimated above, we identified clusters corresponding to African-American ancestry (42 samples, 24% of cohort), European ancestry (82 sample, 47%), and Asian ancestry (4 samples, 2%), while 48 samples (27%) were outside of these clusters and enriched for individuals who identified as Hispanic, Alaskan native, or mixed ancestry (Figure S1; Table S1). Analyzing the first principal component of ancestry shows ancestry groups were differentially represented across developmental periods (F = 3.4, df = 5, p = 0.006, ANOVA), with post hoc analysis showing a greater representation of African American samples in later developmental periods compared with samples of Hispanic, Alaskan native, or mixed ancestry (p.adj = 0.02, TukeyHSD).

Estimation of biological and technical covariates in RNA-seq data

PCA was performed on the covariance matrix of 23,782 cortically expressed genes in 176 samples. A secondary PCA was performed on the 11,705 Non-transitional genes in 167 samples (excluding Period 1 and 2 samples) to assess the extent to which removing the late-fetal transition accounted for variance in gene expression. For each PCA, the variance explained by each principal component was assessed (Figure S2).

To quantify the relative contributions of biological and technical covariates, we calculated the partial R^2 of each covariate with each principal component using the rsq R package, in a generalized linear model where loadings of principal components are considered as a response and biological and technical covariates (such as developmental period, sex, sequencing batch, sequencing depth, RNA integrity number (RIN), mitochondrial RNA proportion, ribosomal RNA proportion, intronic reads proportion, intergenic reads proportion) are considered as predictor variables (Figure S2).

Comparison between BrainVar and BrainSpan

While the BrainVar bulk tissue RNA-seq dataset catalogs gene expression in the DLPFC of 176 donors across development (6 post-conception weeks to 20 years of age), BrainSpan catalogs gene expression across 16 brain regions, including DLPFC, in 40 brains ranging from 8 post-conception weeks to 40 postnatal years (Li et al., 2018). Of note, data were generated for 14 brains in both BrainVar and BrainSpan. To compare the data from the 14 samples profiled in both BrainSpan (Li et al., 2018) and BrainVar, we re-processed the entire BrainSpan dataset using the BrainVar RNA-seq analysis pipeline, transforming raw read counts to $\log_2$CPM values. We then filtered to genes that passed BrainVar minimum expression criteria and calculated sample-to-sample and gene-to-gene Pearson correlation coefficients. We performed principal component analysis on a covariance matrix of the 14 commonly sequenced samples and the 23,782 BrainVar-expressed genes for both datasets. Despite differences in library preparation, with BrainSpan using poly-A priming compared to TruSeq random priming in BrainVar, gene expression was highly correlated per-sample and per-gene .

BrainSpan also generated ChIP-seq datasets of histone 3 lysine 27 acetylation (H3K27ac), a marker of active genes, in DLPFC samples (Li et al., 2018). These H3K27ac data mirror the expression profiles in BrainVar, with Falling genes enriched for fetal-biased H3K27ac peaks and depleted for adult-biased H3K27ac peaks, while Rising genes show the opposite pattern of enrichment (Figure S2).

Transcriptome temporal trajectory estimation

Statistically, the temporal dynamics of the expression of genes can be modeled as a mixture of K distinct trajectories, each with Gaussian noise (Jones et al., 2001; Roeder et al., 1999). To delineate the temporal dynamics for K groups of genes, we used the Flexmix R package, which provides the expected trajectory for each group and the soft group assignments of individual genes to groups.

The expression of 23,782 genes was transformed as $\log_2$(CPM) and normalized by the interquartile range. The samples in epoch 0 were excluded to avoid biased estimation due to very few samples. To identify the overall trend of expression over age, first we fitted the model on all the 23,782 genes assuming there are three groups and that the expected trajectories for each group can be represented with degree-4 polynomials on age. Three typical trajectories were identified, including a group of 6,941 genes with Rising expression levels, a group of 5,173 with Falling expression levels, and a group of 11,705 genes with roughly flat (fitted) expression over time, which we called Non-transitional (Figure 2B).

We considered whether fewer or more trajectory groups described the data better. If we input more than four groups, adjacent classes are automatically combined due to the estimated priors falling below the minimum threshold leaving a maximum of four groups. Using the Akaike information criterion (AIC) and Bayesian information criterion (BIC) we observe the lowest values, indicating a better fit to the data, for four groups, followed closely by three groups, but not for two groups. However, the additional group does not reveal a new profile (e.g., genes rising to period 8/9 then falling for 10-12), instead it distinguishes two Rising groups based on gene variance. Since our objective was to identify temporal gene trajectories, not differences in variance, we selected three groups for the final model to avoid overfitting and ensure interpretability.

Gene ontology functional enrichment for temporal trajectories

For functional enrichment, we characterized genes sets for each trajectory using the R package, gProfiler (Reimand et al., 2011). The pathway enrichment test was performed using Gene Ontology Biological Process terms, which contain between 10 and 2,000 genes, and all 23,782 cortically expressed genes were used as background. Enrichment tests were subject to the “moderate” hierarchical filtering parameter, and the FDR multiple correction in the gProfiler (Figure 2F).

Assessing enrichment in tissue-specific genes from GTEx

To assess enrichment across tissues, 27,546 transcripts with an RPKM $\geq$ 0.5 in 80% of samples one or more tissues in GTEx (https://gtexportal.org/home/) were defined and log-transformed ($\log_2$[RPKM+1]). For each gene, expression between each tissue and all other tissues was assessed using a moderated t-test (R package limma), with models adjusted for age, RIN, gender, and surrogate variables. The Benjamini and Hochberg method was used to estimate false discovery rate (FDR) and tissue enriched genes were defined as: log fold-change > 0.5 and FDR < 0.05. The enrichment of the Falling, Non-transitional, and Rising genes was assessed with using Fisher's exact test with 23,782 cortically expressed genes as a background (Figure 2).

Identifying genes enriched in cell types from single cell data

To identify cell type-enriched genes we calculated a tau metric (Kryuchkova-Mostacci and Robinson-Rechavi, 2017) from the per gene $\log_2$TPM of $\log_2$UMI values for genes within cell type clusters in prenatal forebrain (Nowakowski et al., 2017) and postnatal cortex (Li et al., 2018). Each gene was also ranked across all clusters within the dataset on the basis of TPM/UMI, so that the cluster with the highest TPM/UMI was ranked as "1," while the cluster with the second highest TPM/UMI for that gene was ranked as "2," etc. The genes were sorted by TPM/UMI rank and visually inspected for cell type specificity or substantial enrichment in single cell RNA-seq data from the prenatal and postnatal human cortex (https://cells.ucsc.edu/?ds=cortex-dev#). For each cell type, the top ten genes that showed clear specificity/enrichment were selected.

Enrichment of gene trajectories in temporal putative cis-regulatory elements

H3K27ac peaks present in more than two samples of fetal or adult dorsal frontal cortex in BrainSpan (Li et al., 2018) were tested for fetal versus adult temporal bias using DESeq2 (Love et al., 2014). Temporally biased genes were defined as adjusted $p < 0.01$ and fold change $\geq$ 2. A category of "non-temporal" H3K27ac peaks was generated with peaks showing $p > 0.05$. All peaks were annotated using the gene with the closest transcription start site in Gencode v21. Genes were classified as only-fetal or only-adult if they were associated with fetal or adult-only H3K27ac peaks, respectively. Enrichments of each category of H3K27ac-genes in each category of eGenes were tested by means of a Fisher Exact's test and p-values were adjusted using Benjamini-Hochberg, using genes associated to non-variant H3K27ac peaks as a reference background.

WGCNA network construction and module definition

To assess the functional topology in cortical samples, we applied Weighted Gene Co-Expression Network Analysis (WGCNA) (Langfelder and Horvath, 2008) to 23,782 cortically expressed transcripts. Network analysis was performed with WGCNA (version 1.63) using a signed network, choosing a soft-threshold power, the mean connectivity less than 50, and scale-free topology greater than 0.8. To reduce the bias driven by a few sample outliers, we applied the blockwiseConsensusModules function, which detects consensus modules across 100 subsampled networks. We used the average linkage hierarchical clustering of the topological overlap dissimilarity matrix (1-TOM) to generate the network dendrogram. Modules were defined as branches of the dendrogram using the hybrid adaptive tree cut with the following parameters: minimum module size = 200, negative pamStage, height cut = 0.999, and deep split = 2 (Langfelder and Horvath, 2007). Modules were summarized by their first principal component (ME, module eigengene), followed by merging modules with high correlations (eigengene value $\geq$ 0.9).

WGCNA functional enrichment for module characterization

For functional enrichment, we characterized WGCNA module genes using the gProfiler R package (Reimand et al., 2011), as described above in the analysis of temporal trajectory genes. To identify WGCNA module genes that are regulatory targets, we searched for transcription factor binding targets using the ChEA (Lachmann et al., 2010; Satterstrom et al., 2020), and TRANSFAC (Matys et al., 2003), and microRNA using the mirTarbase database (Chou et al., 2018).

WGCNA module preservation

To assess whether 19 co-expression modules in our samples were preserved in other, independent DLPFC or frontal cortex expression datasets, we compared our dataset with non-overlapping samples from the BrainSpan dataset (Li et al., 2018) and applied the module preservation function from the WGCNA R package (Langfelder et al., 2011). From the BrainSpan dataset, we selected DLPFC (n = 30), 10 non-DLPFC neocortical regions (n = 317), and subcortical regions excluding the cerebellum (n = 140). Given the original co-expression network constructed above, we used modulePreservation to calculate module preservation statistics from 100 permutations (Table S3).

Clustering analysis in protein-protein interaction network

To examine functional association of a group of genes/proteins, we performed a clustering analysis of a protein-protein interaction network implemented in SANTA R package (Cornish and Markowetz, 2014). Ripley's K function provides a measure of whether points are clustered together or randomly dispersed (homogeneous) in a network and the SANTA R package reformulated Ripley's K function for a protein-protein interaction network. Clustering can be indicative of functional association between the genes/proteins under consideration. To test departure from homogeneity of a given gene set, we drew an empirical null distribution of clustering from 1,000 random samples of matching sized gene sets from the BioGRID protein-protein interaction network data (Stark et al., 2006;

Winter et al., 2011) (v3.4.132). We reported departure from null distribution as a Z-score (i.e., $Z > 0$: level of clustering of a gene set greater than null expectation and $Z < 0$: level of clustering of a gene set less than null expectation).

Cis-eQTL detection and classification

Cis-eQTLs were identified for all high quality, common variants (N = 6,573,196) within 1 Mb of a gene boundary using the linreg function in Hail 0.1, with period, sex, and the first five principal components of common variant ancestry as covariates. This analysis was run on three cuts of the BrainVar dataset: complete sample (N = 176, periods 1-12), prenatal-only (N = 112, periods 1-6), and postnatal-only (N = 60, periods 8-12). Separately for the results of each analysis, false discovery rate (FDR) was calculated for all gene-variant pairs using the Benjamini-Hochberg procedure.

We then classified all gene-variant pairs with FDR $\leq$ 0.05 from at least one analysis into groups defined by the temporal specificity of their eQTL effects. To do this, we first identified one variant per gene with the smallest, FDR-significant p value, from any of the three analyses. We then used a Z-test to compare the regression coefficients for these variant-gene pairs from the prenatal and postnatal analyses:

$$Z = \frac{\beta_{\mathrm{Pre}} - \beta_{Post}}{\sqrt{SE^2_{\mathrm{Pre}} + SE^2_{Post}}}$$

Using the results from the eQTL analyses and from this prenatal-postnatal comparison, we then classified each of these top (one per gene) gene-variant pairs and their corresponding target gene (eGene) into one of five groups:

1. Constant eQTLs/eGenes, characterized by consistent effects across this developmental dataset: FDR $\leq$ 0.05 in the complete sample analysis, same direction of effect and unadjusted p $\leq$ 0.05 in both the prenatal and postnatal analyses
2. Prenatal-predominant eQTLs/eGenes, with strongest effects during prenatal development: FDR $\leq$ 0.05 in the prenatal analysis, unadjusted p > 0.05 in the postnatal analysis, pre-post comparison Z-test FDR-adjusted p $\leq$ 0.05
3. Postnatal-predominant eQTLs/eGenes, with strongest effects during postnatal development: FDR $\leq$ 0.05 in the postnatal analysis, unadjusted p > 0.05 in the prenatal analysis, pre-post comparison Z-test FDR-adjusted p $\leq$ 0.05
4. Prenatal-trending eQTLs/eGenes, which did not fit into earlier categories, but had higher prenatal effects ($B_{\mathrm{Pre}} > B_{\mathrm{Post}}$)
5. Postnatal-trending eQTLs/eGenes, which did not fit into earlier categories, but had higher prenatal effects ($B_{\mathrm{Post}} > B_{\mathrm{Pre}}$)

All FDR-significant variants associated with the expression of a single gene were classified into one of these five groups according to the classification of the top variant for the same gene (Figure 5).

Alternative approaches for assigning eGenes to temporal categories

Many eGenes are associated with multiple eQTLs, each of which could individually meet criteria for any one of the five temporal categories. As described above, we categorized eGenes into temporal categories based on the performance of their top eQTL (smallest p-value), but we assessed the performance against three alternative approaches: (1) eGene assigned to the same category as a majority of their eQTLs (“majority eQTL” approach), with ties assigned in the order Constant, Prenatal-predominant, Postnatal-predominant, Prenatal-trending, Postnatal-trending, (2) for eGenes with $\geq$ 1 eQTL, category assignment based on the performance of the second most significant variant (“second eQTL” approach), and (3) each eQTL individually assigned to categories (“individual eQTL” approach). For each of these alternative approaches, we calculated the percent of eGenes or eQTLs from each top variant-based category that were assigned to each category using the majority eQTL, the second eQTL, or the individual eQTL approach (Figure S5).

Assessment of ancestry differences in prenatal and postnatal sample sets using genomic control

To validate that our eQTL discovery analyses were adequately adjusted for differences in sample ancestry, we calculated genomic control, or lambda, an estimate of inflation of genetic association signal (Devlin and Roeder, 1999), from the results of each of the three analyses (complete sample, prenatal-only, postnatal-only). To do this, we randomly sampled 500 gene-variant pairs that occur on the same chromosome but are located 10-100 Mb apart, under the assumption that these distant gene-variant pairs would be unlikely to be enriched for true gene expression association. Using the eQTL signals for these 500 gene-variant pairs from the three analyses, we calculated lambda for analysis. We repeated this procedure for 100 random selections of 500 distant gene-variant pairs. We find that the median lambda values across 100 permutations for each are near 1, in keeping with a test has been properly corrected for population structure (specifically, 1.01 for prenatal, 0.99 for postnatal, and 1.00 for the complete sample).

Comparison with published eQTL studies

To assess the sensitivity of our cis-eQTL discovery analysis relative to previous work, we evaluated the relationship between sample size and eGene discovery for: the BrainVar prenatal, postnatal, and complete sample analyses as run using the HCP- and SVA-adjusted expression data, GTEx v7 analyses by tissue (gtexportal.org), postnatal human frontal cortex by the CommonMind Consortium (Fromer et al., 2016), and prenatal human whole brain (O’Brien et al., 2018). Using the sample size reported in each analysis, or for each tissue (GTEx), and the number of genes with at least one eQTL reaching significance of FDR $\leq$ 0.05, we plotted

the relationship between eGene discovery and sample size (Figure S5). We observe a strongly positive relationship across BrainVar and the published analyses, in keeping with prior reports that eQTL and eGene discovery is positively associated with sample size (Aguet et al., 2017).

We evaluated the performance of the eQTLs that we identified in our analyses with published sets of eQTLs identified in the human postnatal frontal cortex (Aguet et al., 2017) and in the human prenatal whole brain (O'Brien et al., 2018). For the postnatal frontal cortex data, we downloaded significant variant-gene pairs from the GTEx v7 data release from gtexportal.org and used R to write out the variant locations to a bed file format. We then used the command line LiftOver utility from the UCSC Genome Browser to convert the hg19 variant positions to GRCh38. For the prenatal brain data, we downloaded the eQTL summary statistics, results for the top eQTLs per gene, and SNP positions bed file from the study data repository on Figshare (https://figshare.com/articles/Summary_statistics_for_expression_quantitative_trait_loci_in_the_developing_human_brain_and_their_enrichment_in_neuropsychiatric_disorders/6881825). We then used each gene's nominal significance threshold from the top eQTLs file to identify the full set of variant-gene pairs meeting significance.

Using variant (GRCh38 position, reference, and alternate alleles) and gene (Ensembl gene IDs) identifiers, we matched significant variant-gene pairs separately from GTEx frontal cortex and prenatal brain to the variant-gene pairs meeting FDR significance (≤ 0.05) in the BrainVar analyses. For all significant eQTLs in BrainVar that overlapped with the reference datasets, we then compared the effect of the variant on the expression of its associated gene to determine the percentage of overlapping eQTLs with concordant direction of effect, as well as the Pearson correlation between the eQTL effects (beta from BrainVar, slope from GTEx or prenatal whole brain). The significance of this correlation was evaluated using the cor.test function in R.

Distance between eQTLs and transcription start site

The distance between each significant eQTL and the transcription start site (TSS) of its associated eGene was calculated by comparing the variant position to the TSS position and strand of the gene according to Gencode v21. Positive distances indicate variants downstream of the TSS, negative distances indicate upstream variants. Comparison between groups of eQTLs was run using only the top eQTL per gene and the absolute value of distance from the associated gene's TSS.

Overlap of eQTLs with H3K27ac

We tested the global overlap between eQTLs and H3K27ac from human fetal, infant and adult dorsal frontal cortex and cerebellum and embryonic cortex from BrainSpan (Li et al., 2018) and Reilly et al. (2015). Intersection between sets of coordinates were performed using Bedtools (Quinlan and Hall, 2010). We tested three sets of variants: (1) best eQTL per eGENE, (2) all significant eQTLs with FDR < 0.05 in the corresponding eQTL category of Prenatal-predominant, Postnatal-predominant or Constant, and (3) a background group composed by all variants tested for eQTL, excluding those with a $p < 0.05$ to any gene at any time period tested. 95% confidence intervals were obtained by bootstrapping variants 100 times (Figure S5).

Enrichment of eQTLs in functional genomic elements

We tested the enrichment of different categories of eQTLs in sets of genomics elements using GREGOR (Schmidt et al., 2015). We tested all best eQTL per gene in: (1) dorsal frontal cortex H3K27ac peaks from fetal and adult brain samples, and (2) 18 chromatin states whole-genome segmentation of sample E073-Medial Frontal Cortex Lobe from The Roadmap Epigenomics Project (Kundaje et al., 2015). We reported observed/expected number of overlapping eQTLs and BH adjusted p-values (Figure S5).

Test for differential expression variance in prenatal and postnatal stages

We used an F test to compare prenatal variance (periods 1-6) and postnatal variance (periods 8-12) in gene expression level, correcting for age (period), sex, and five ancestry principal components within each stage. At a Benjamini-Hochberg-adjusted p value ≤ 0.05, we identified 8,094 genes with greater prenatal variance, and 1,752 genes with greater postnatal variance.

Gene sets associated with CNS traits and disorders

To compare with 23,782 cortically expressed genes, we created the list of gene sets for previous trait and disorder association and functional properties. Gene identifiers were converted between studies based on the complete HUGO Gene Nomenclature Committee dataset. Autism spectrum disorder (ASD) risk genes were obtained from Satterstrom et al. (2020), an exome sequencing based gene discovery refining to high-confidence genes (n = 99) at a false discovery rate (FDR) ≤ 0.1. Genes associated with developmental delay (n = 93) were selected from the exome sequencing analysis of the Deciphering Developmental Disorders project (Deciphering Developmental Disorders Study, 2017). From Heyne et al. (2018), we chose 33 genes as high-confidence epilepsy candidates, where multiple *de novo* variants were seen.

We used significant loci from the genome-wide association studies (GWAS) of attention deficit hyperactivity disorder (ADHD) (Demontis et al., 2019), Alzheimer's disease (Lambert et al., 2013), educational attainment (Lee et al., 2018), schizophrenia (Schizophrenia Working Group of the Psychiatric Genomics Consortium, 2014), major depressive disorder (Wray et al., 2018), multiple sclerosis (Beecham et al., 2013), Parkinson's disease (Chang et al., 2017). We selected loci from a summary statistics file if publicly available, otherwise we used the table of genome-wide significant loci from each study. We retrieved genes harboring loci or within 10kb from loci. We excluded the extended major histocompatibility complex region on the chromosome 6, known to have a

substantial number of genes due to high linkage disequilibrium (Schizophrenia Working Group of the Psychiatric Genomics Consortium, 2014) for downstream enrichment analyses.

Constrained genes were defined as probability of loss-of-function intolerant (pLI) score $\geq$ 0.995 in the ExAC database (Lek et al., 2016). Genes specific to cell types in the mid-fetal cortical development were selected from Nowakowski et al. (2017) and BrainSpan (Li et al., 2018). For all gene lists, see Table S2 and Table S4.

Enrichment of DLPFC eQTLs in SNPs associated with complex phenotypes

We tested for enrichment of DLPFC eQTLs among GWAS significant SNPs using a permutation-based procedure. GWAS SNPs were taken from published GWAS summary statistics at a significance threshold of $p < 5 \times 10^{-8}$. The following procedure was repeated separately for summary statistics from GWAS of four phenotypes: schizophrenia (Schizophrenia Working Group of the Psychiatric Genomics Consortium, 2014), educational attainment (Lee et al., 2018), multiple sclerosis (Beecham et al., 2013), and Alzheimer's disease (Lambert et al., 2013). First, SNPs in the summary statistics and the list of SNPs tested for eQTL discovery were filtered to those in 1000 Genomes Project data. SNPs in the GWAS summary statistics were then filtered to variants also tested for eQTL discovery, using direct (same SNP) or proxy ($r^2 > 0.8$ in CEU 1000 Genomes samples) as defined by the PLINK 1.9 – r2 command (Chang et al., 2015a). To estimate the proportion of eQTL SNPs among phenotype associated SNPs versus among all SNPs (or a sample of null SNPs), we recognized three important factors that could differ between null SNP sets and the phenotype-associated SNPs: LD structure, MAF distribution, and gene density. First, to account for LD structure, we used PriorityPruner version 0.1.4 (http://prioritypruner.sourceforge.net) to LD prune SNPs supervised by GWAS p-value in order to preferentially retain as many phenotype-associated SNPs as possible while adequately removing SNPs in high LD ($r^2 > 0.7$ within a sliding 500kb window) using in 1000 Genomes CEU sample data. Second, the remaining SNPs were grouped according to their MAF decile. Third, each remaining SNP was grouped into decile of gene density to allow for differential opportunity to be identified as an eQTL. Gene density was determined by the number of genes within the 1MB eQTL detection window as defined by the annotation package "TxDb.Hsapiens.UCSC.hg38.knownGene" from R Bioconductor (Huber et al., 2015). SNPs in the ~3.2 MB HLA region (hg38 coordinates: chr6:29,751,784-32,915,731) as defined by the "GWASTools" R Bioconductor package (Gogarten et al., 2012) and UCSC genome browser (Kent et al., 2002) were excluded from enrichment testing. Next, 1 million null SNP sets were drawn by matching each phenotype-associated SNP (GWAS $p < 5 \times 10^{-8}$) to a random SNP matched on both MAF and gene density. Enrichment fold statistics were computed as the proportion of eQTLs in the phenotype-associated set divided by the mean proportion of eQTLs across null sets. P values were calculated as the proportion of null set fold-enrichment statistics as or more extreme than the observed phenotype-associated fold enrichment statistic. This permutation procedure was repeated for each of six eQTL SNP lists: all, Constant, Prenatal-predominant, Prenatal-trending, Postnatal-predominant, and Postnatal-trending.

Gene-set analysis of eGenes and GWAS data

To assess whether eQTL targets (eGenes) are enriched for GWAS signal, we performed competitive gene set enrichment analysis for each group of eGenes using the MAGMA software (de Leeuw et al., 2015). We input the eGene lists from Prenatal-predominant, Postnatal-predominant, Constant, Prenatal-trending, and Postnatal-trending (Table S5) and summary statistics from published GWAS of schizophrenia (Schizophrenia Working Group of the Psychiatric Genomics Consortium, 2014), autism spectrum disorder (Grove et al., 2019), educational attainment (Lee et al., 2018), multiple sclerosis (Beecham et al., 2013), Alzheimers disease (Lambert et al., 2013), triglycerides (Willer et al., 2013), and height (Wood et al., 2014). First, SNPs from the GWAS summary statistics files were annotated to NCBI protein-coding genes that passed RNA-seq QC in our DLPFC expression data with a 10kb window on either side of the gene boundaries. Next, a gene-level analysis was done to determine the strength of association for each gene with phenotype of interest. To assess whether genes in the eGene gene-sets are more strongly associated with the phenotype of interest than other genes, gene-based z-scores are regressed on a gene-set indicator variable and MAGMA default covariates (gene size, gene density, sample size, 1/MAC, and the log of each of these). The beta coefficient for the gene-set indicator variable is tested for significance $H_A : \beta_1 > 0$. Results from this analysis are reported in Table S6. We did not observe significant enrichment of GWAS signal from any of the six phenotypes tested in any of the five temporally assigned eGene gene sets. However, we repeated a similar test, annotating GWAS summary statistics SNPs to NCBI protein-coding genes with 10kb flanking region that passed RNA-seq QC in our DLPFC expression data and had an assigned pLI score, and found that a gene-set defined as pLI score > = 0.995 showed significant enrichment for stronger GWAS association in all tested phenotypes except for multiple sclerosis, compared to genes with pLI score < 0.995.

Co-localization analysis of CNS traits and disorders

Coloc (Giambartolomei et al., 2014) was used to formally test for co-localization of GWAS signal from schizophrenia (Schizophrenia Working Group of the Psychiatric Genomics Consortium, 2014) and educational attainment (Lee et al., 2018) summary statistics with our five eQTL categories. Coloc tests five hypotheses (H0: no association, H1: GWAS association only, H2: eQTL association only, H3: both but not co-localized, H4: both and co-localized) and returns a posterior probability for each hypothesis in each region. Posterior probably of H4 > = 0.8 is strong Bayesian evidence of co-localization.

DATA AND CODE AVAILABILITY

Open source scripts used in this study are referenced throughout. The pipeline for whole-genome analysis is available online at: https://github.com/sanderslab/psychcore-compute-platform. The accession number for the raw RNA-seq and WGS data reported in this paper, along with processed files, is PsychENCODE Knowledge Portal: syn21557948 on Synapse.org (https://www.synapse.org/#!Synapse:syn4921369).

Cell Reports, Volume *31*

Supplemental Information

Whole-Genome and RNA Sequencing Reveal Variation and Transcriptomic Coordination in the Developing Human Prefrontal Cortex

Donna M. Werling, Sirisha Pochareddy, Jinmyung Choi, Joon-Yong An, Brooke Sheppard, Minshi Peng, Zhen Li, Claudia Dastmalchi, Gabriel Santpere, André M.M. Sousa, Andrew T.N. Tebbenkamp, Navjot Kaur, Forrest O. Gulden, Michael S. Breen, Lindsay Liang, Michael C. Gilson, Xuefang Zhao, Shan Dong, Lambertus Klei, A. Ercument Cicek, Joseph D. Buxbaum, Homa Adle-Biassette, Jean-Leon Thomas, Kimberly A. Aldinger, Diana R. O'Day, Ian A. Glass, Noah A. Zaitlen, Michael E. Talkowski, Kathryn Roeder, Matthew W. State, Bernie Devlin, Stephan J. Sanders, and Nenad Sestan

Supplemental Information

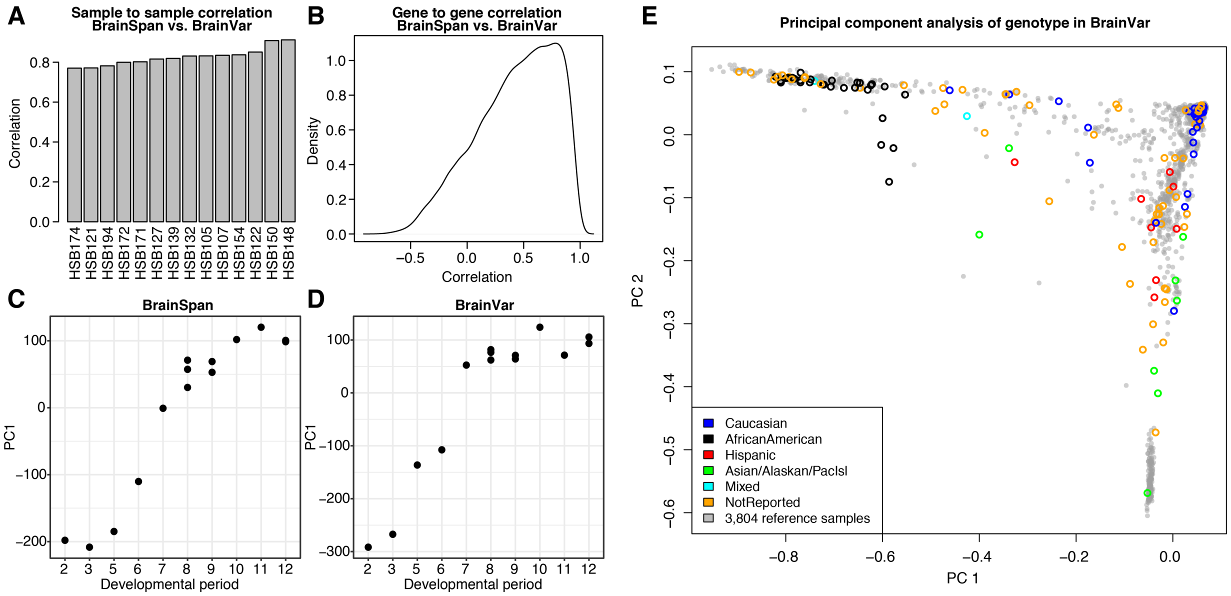

Figure S1. Comparison of RNA-seq data between BrainSpan and BrainVar and WGS ancestry prediction, related to Figures 1, 2. A) Pearson correlation coefficients of gene expression for 14 overlapping samples with RNA-seq data generated from the dorsolateral frontal cortex in BrainSpan (Li et al., 2018) and BrainVar. **B)** Distribution of Pearson correlation coefficients between 23,782 cortically expressed genes for RNA-seq data for 14 overlapping samples in BrainSpan (Li et al., 2018) and BrainVar. **C)** First principal component of gene expression by developmental period for all samples in the BrainSpan dataset (Li et al., 2018). **D)** First principal component of gene expression by developmental period for all samples in BrainVar. **E)** Principal component analysis using common variation called from WGS data for all 176 samples in BrainVar against a reference of 3,804 independent parents with WGS data from the Simons Simplex Collection (An et al., 2018). Self-reported ancestry is indicated by color for the BrainVar samples.

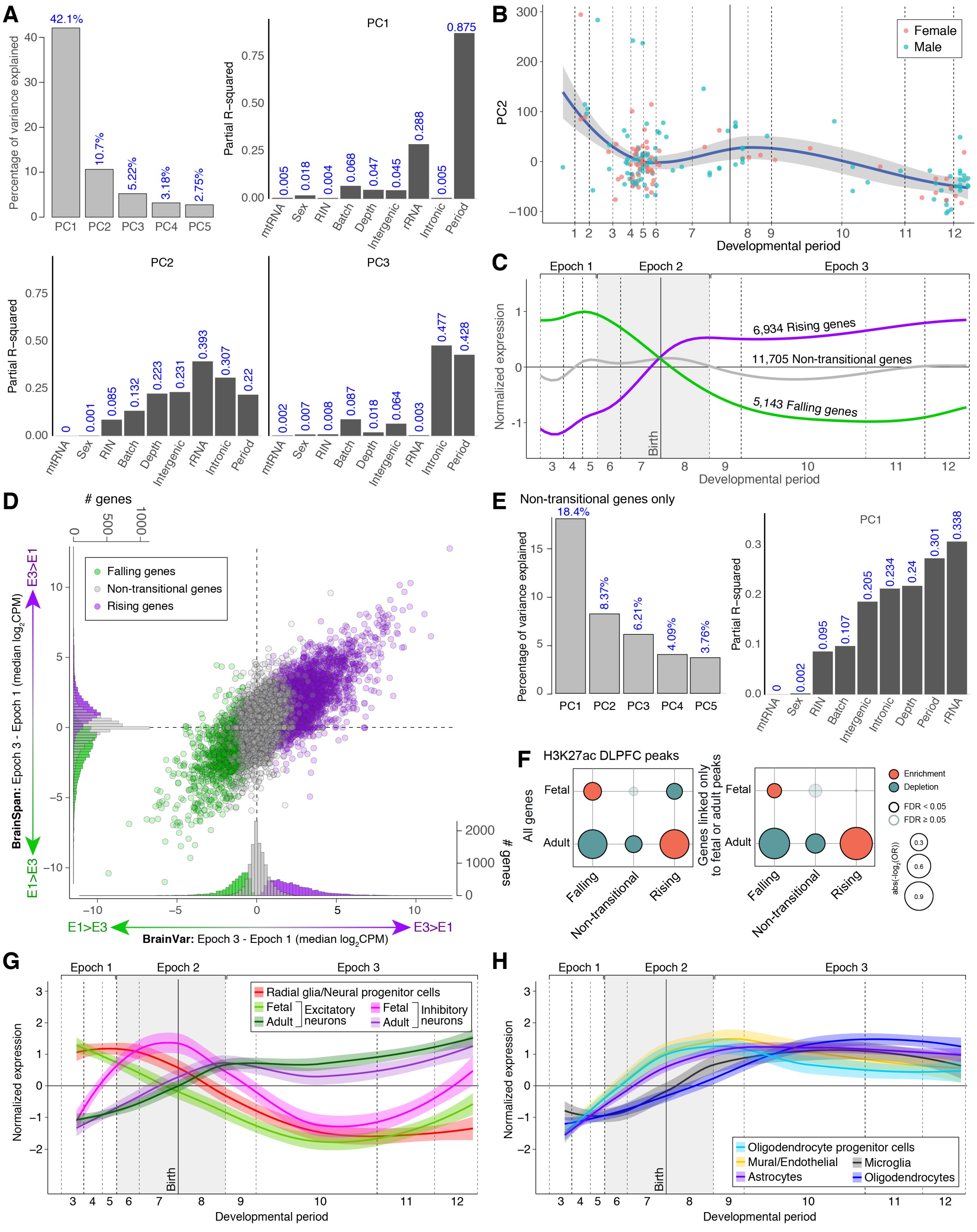

Figure S2. Principal component analysis of gene expression, related to Figure 2. A) A scree plot showing the variance in gene expression explained by the first five principal components (PC1 to PC5) across all samples and 23,782 cortically-expressed genes in the BrainVar dataset. **B)** The relationship between PC2 (y-axis) and

developmental period (x-axis); the equivalent plot for PC1 is shown in Figure 2A of the main manuscript. **C)** Relationship between PC1, PC2, and PC3 for each sample (points) with developmental period indicated by color and genotypic sex by symbol. **D)** Correlation between PC1, PC2, and PC3, known variables, and residuals. **E)** Trajectory analysis identified 12,077 genes involved in the late-fetal transition and 11,705 Non-transitional genes (Figure 2B). To assess the extent to which the late-fetal transition explains the temporal variance captured by PC1 in the initial analysis, we repeated the principal component analysis for the 11,705 Non-transitional genes. PC1 of this secondary analysis explains on 18.4% of the variance in gene expression. **F)** The correlation between PC1 and known variables and residuals for the secondary analysis based on Non-transitional genes only. **G)** The enrichment for H3K27ac peaks, detected in the fetal and adult human dorsolateral frontal cortex in BrainSpan (Li et al., 2018), with Falling, Non-transitional and Rising genes. The analysis is shown for all 23,782 cortically-expressed genes (top) and limited to genes with an associated H3K27ac peak during at least one developmental stage.

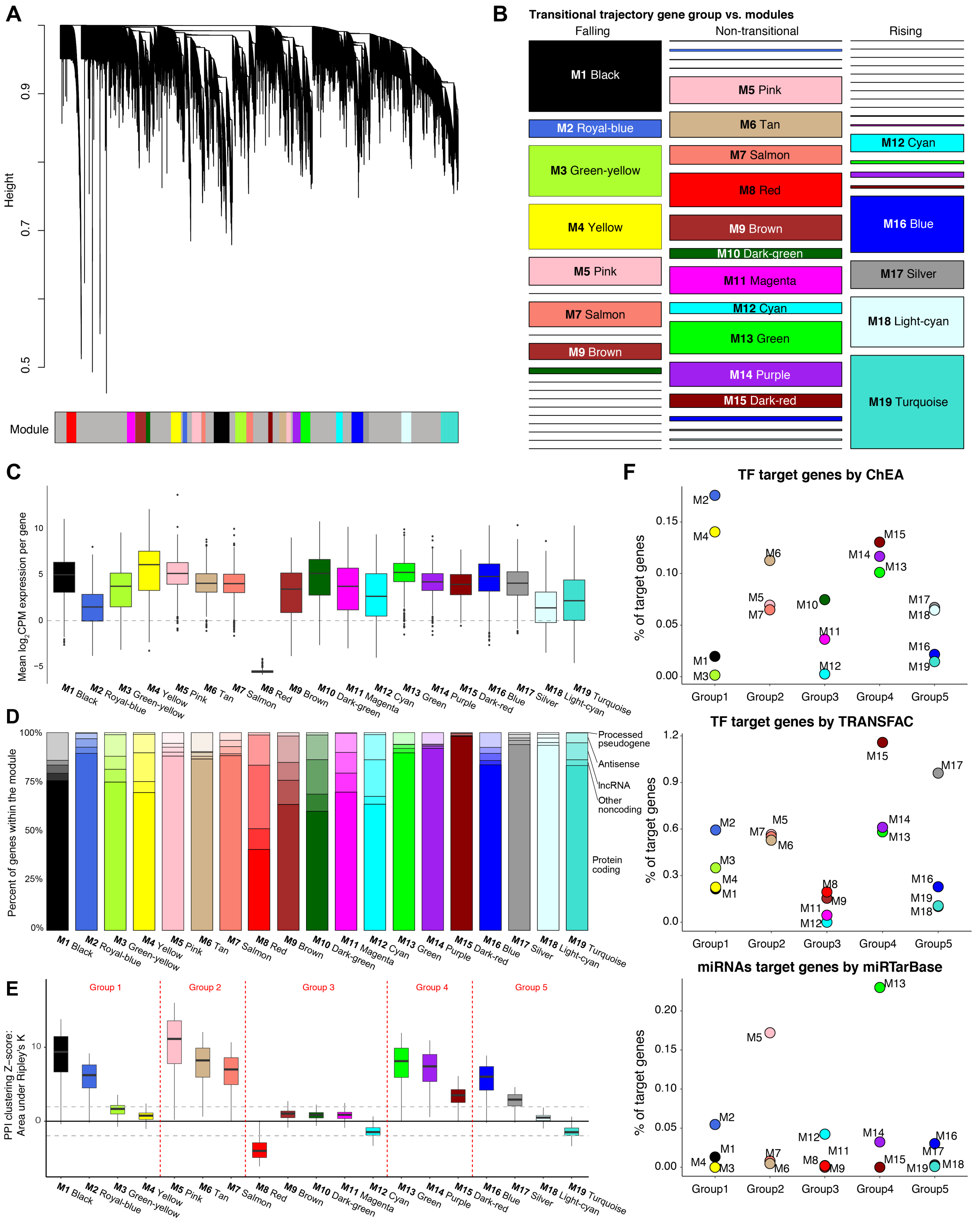

Figure S3. WGCNA module relationship and characteristics, related to Figure 3. A) Dendrogram of 10,459 cortically-expressed genes assigned to 19 WGCNA modules; 13,323 genes were not assigned to any module (grey). **B)** A mosaic plot showing the overlap of genes between modules (y-axis and color) and the three transitional

trajectory gene sets (x-axis), with the area of each rectangle representing the number of overlapping genes. **C)** Median log_2CPM expression of genes within each module (x-axis and color) across all 176 samples in the cohort. **D)** The percentage of protein-coding and noncoding genes (lncRNA, Antisense, Processed pseudogenes, and other noncoding shown by opacity) within each module. **E)** The connectivity within BioGRID protein-protein interaction (PPI) networks of the genes within each module is shown as a Z-score distribution (Cornish and Markowetz, 2014) by permutation against all 23,782 cortically-expressed genes. **F)** The percentage of genes in each module targeted by transcription factors (TFs) predicted by ChIP Enrichment Analysis (ChEA, (Lachmann et al., 2010), top) and TRANSFAC (http://genexplain.com/transfac/, middle) and the percentage of genes targeted by miRNA prediction from the mirTarbase database ((Chou et al., 2018), bottom).

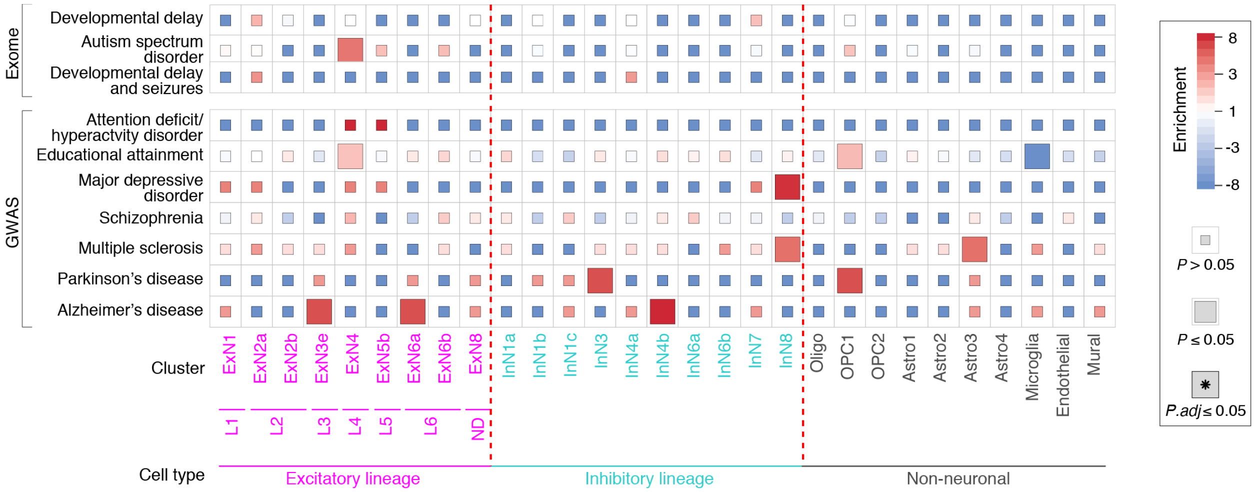

Figure S4. Expression of genes associated with CNS traits and disorders, related to Figure 4. The enrichment of genes associated with ten CNS traits and disorders (Figure 4) is shown in cell type clusters from single-cell sequencing of three adult human brain samples (Li et al., 2018). Abbreviations: C: cluster of single nuclei; L: cortical layer; ND: layer not defined; RG: radial glia; IPC: intermediate progenitor cell; NN: newborn neuron; ExN: excitatory neuron; InN: inhibitory neuron; CGE: caudal ganglionic eminence; MGE: medial ganglionic eminence; OPC: oligodendrocyte progenitor cell. Statistical analysis: FET, corrected for 290 comparisons.

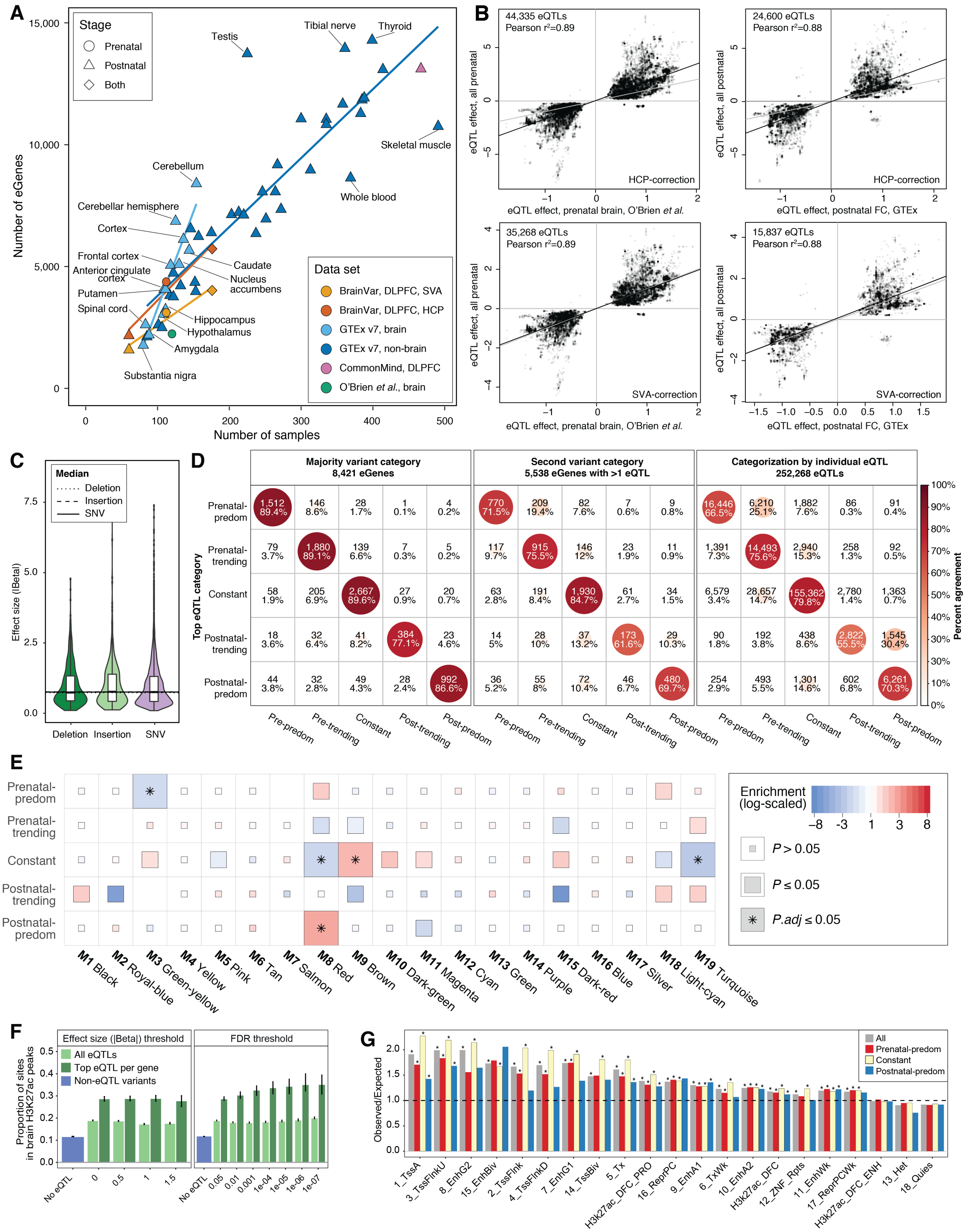

Figure S5. eGene concordance and characteristics, related to Figure 5. A) Scatterplot and best fit lines for the number of eGenes (y-axis) against sample size (x-axis) reported in BrainVar and several published data sets (Fromer

et al., 2016; O'Brien et al., 2018; The GTEx Consortium et al., 2017). **B)** Scatterplot of the eQTL direction and magnitude of effect for variant-gene pairs reaching FDR-significance in BrainVar and published datasets (O'Brien et al., 2018; The GTEx Consortium et al., 2017). The plots on the left compare BrainVar to prenatal whole brain (O'Brien et al., 2018), while those on the right compare BrainVar to adult DLPFC (The GTEx Consortium et al., 2017). The plots at the top are for eQTLs discovered in the BrainVar prenatal samples, while those at the bottom are eQTLs discovered in BrainVar postnatal samples. Best fit line is plotted in black, slope of 1 is plotted in gray. **C)** Distribution of the absolute value of eQTL effect size (regression beta) for all eQTLs binned by deletions, insertions, and single nucleotide variants (SNV). **D)** The temporal predominance of an eGene was defined by the eQTL with the lowest p-value (top eQTL). To assess the consistency of this approach, we assessed the number of eGenes where the temporal predominance of the top eQTL matched the majority of eQTLs for the eGene (left) or the eQTL with the second lower p-value (right). **E)** Enrichment of 19 WGCNA modules with eGenes, divided into five categories based on temporal specificity. **F)** Proportion of eQTLs that overlap an H3K27ac peak detected in human DLPFC in BrainSpan (Li et al., 2018). Results are binned by eQTL effect size (left) and FDR threshold (right) showing all eQTLs and the top eQTL per gene (shown by shade). **G)** Barplot of observed versus expected overlap using GREGOR analysis between Temporal-predominant and Constant eQTLs with functional loci from 18 chromatin states defined by the Roadmap Epigenome Project (https://egg2.wustl.edu/roadmap/web_portal/chr_state_learning.html#exp_18state).

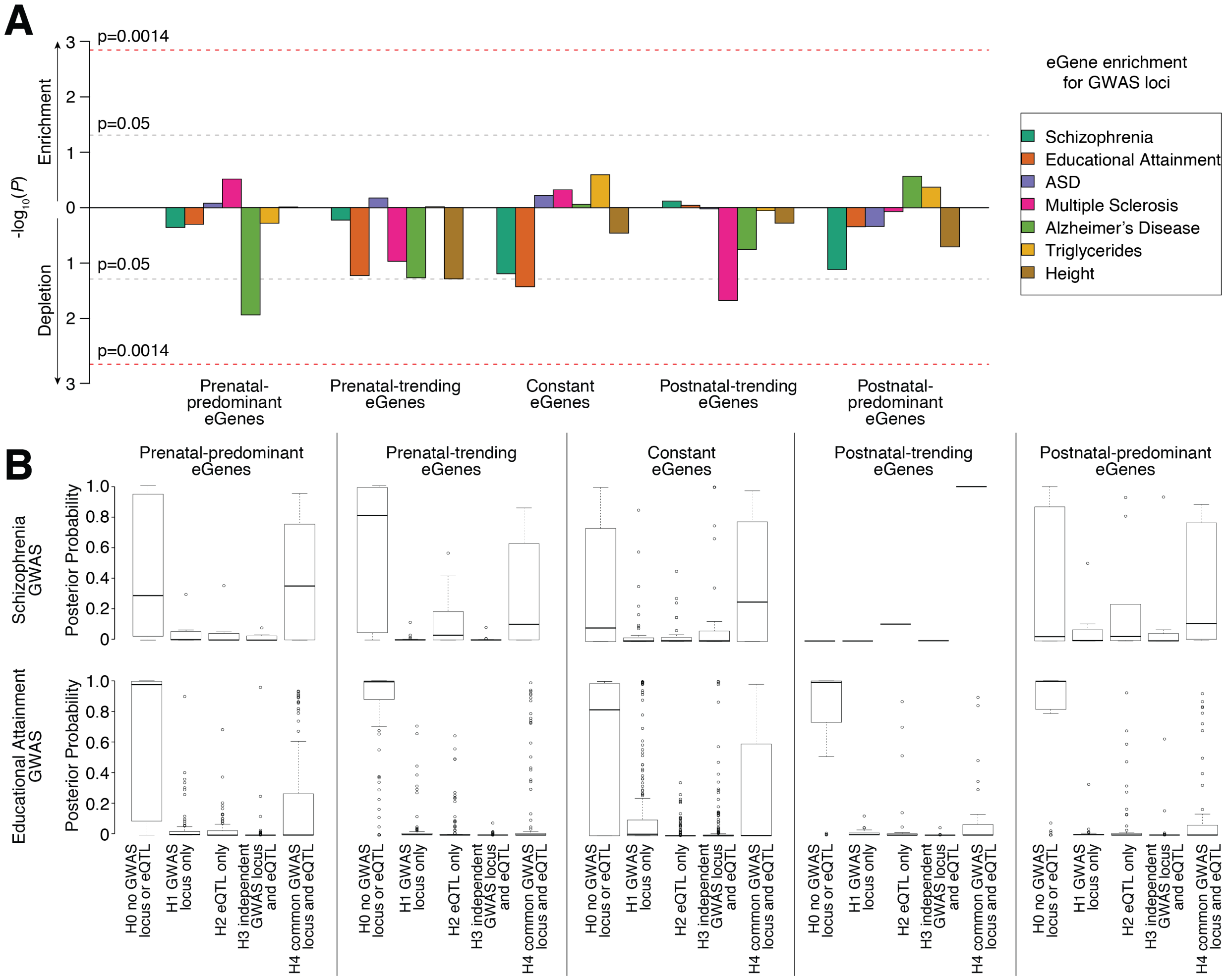

Figure S6. eQTL enrichments and colocalization, related to Figure 6. A) Barplot of enrichment p-values for GWAS signal in temporal categories of eGenes using MAGMA analysis. Red dashed line indicates significance threshold after Bonferroni correction for tests of 7 GWAS phenotypes and 5 eGene categories. **B)** Boxplots of posterior probabilities (PPs) for tests of colocalization between eQTL and GWAS loci. For each tested region, the PP is shown for the 5 hypotheses tested using Coloc analysis software, grouped by temporal category of the eGenes associated with the eQTL loci.
